# Dynamic Pattern of HOXB9 Protein Localization during Oocyte Maturation and Early Embryonic Development in Mammals

**DOI:** 10.1371/journal.pone.0165898

**Published:** 2016-10-31

**Authors:** Caroline Sauvegarde, Delphine Paul, Laure Bridoux, Alice Jouneau, Séverine Degrelle, Isabelle Hue, René Rezsohazy, Isabelle Donnay

**Affiliations:** 1 Biologie Moléculaire et Cellulaire Animale (AMCB), Institut des Sciences de la Vie (ISV), Université catholique de Louvain, Louvain-la-Neuve, Belgium; 2 UMR BDR, INRA, ENVA, Université Paris Saclay, Jouy-en-Josas, France; 3 Institut National de la Santé et de la Recherche Médicale (INSERM), UMR-S1139, U767, Faculté des Sciences Pharmaceutiques et Biologiques, Paris, France; 4 Université Paris Descartes, Sorbonne Paris Cité, Paris, France; 5 PremUp Foundation, Paris, France; Michigan State University, UNITED STATES

## Abstract

**Background:**

We previously showed that the homeodomain transcription factor HOXB9 is expressed in mammalian oocytes and early embryos. However, a systematic and exhaustive study of the localization of the HOXB9 protein, and HOX proteins in general, during mammalian early embryonic development has so far never been performed.

**Results:**

The distribution of HOXB9 proteins in oocytes and the early embryo was characterized by immunofluorescence from the immature oocyte stage to the peri-gastrulation period in both the mouse and the bovine. HOXB9 was detected at all studied stages with a dynamic expression pattern. Its distribution was well conserved between the two species until the blastocyst stage and was mainly nuclear. From that stage on, trophoblastic cells always showed a strong nuclear staining, while the inner cell mass and the derived cell lines showed important dynamic variations both in staining intensity and in intra-cellular localization. Indeed, HOXB9 appeared to be progressively downregulated in epiblast cells and only reappeared after gastrulation had well progressed. The protein was also detected in the primitive endoderm and its derivatives with a distinctive presence in apical vacuoles of mouse visceral endoderm cells.

**Conclusions:**

Together, these results could suggest the existence of unsuspected functions for HOXB9 during early embryonic development in mammals.

## Introduction

HOXB9 is a homeodomain transcription factor of the HOX family which is well conserved within the animal kingdom. In mammals, there are 39 *HOX* genes organized into four chromosomal complexes (A, B, C and D) and defining 13 groups of paralogues numbered from 1 to 13. From the gastrulation stage onward, HOX proteins are known to be involved in the patterning of the anterior-posterior axis of the embryo, in limb development and in organ formation [1–4]. They have multiple functions in cell proliferation, specification and death (reviewed in [5, 6]). Besides their role as regulators of gene expression, they are involved in non-transcriptional functions such as DNA replication, DNA repair and mRNA translation (reviewed in [7]). HOXB9, in particular, takes part in the formation of the rib cage and contributes to forelimbs development [4, 8, 9]. Homozygous mice present abnormalities of the sternum, fusion of the anterior ribs and attachment of the eight ribs to the sternum. In the adult, it is involved in blood cell differentiation [10] and development of the mammary epithelium during gestation and lactation [11].

As for most *Hox* genes, the expression pattern of *Hoxb9* has been well-described in the mouse, from the gastrulation stage on, and paralleled to its roles in patterning the main body axis and the forelimbs. After gastrulation, mouse *Hoxb9* mRNA are detected first at the early headfold (EHF) stage, in the primitive streak and adjacent mesoderm, while no expression is detected in late allantoic bud (LB) stage [12–14]. During the course of embryogenesis, *Hoxb9* is expressed in the neural tube as well as in the paraxial mesoderm and its derivatives. The most anterior limit of expression in the neural tube is reached at embryonic day 10.5 (E10.5) at the level of somite 6 (first cervical somite—[8]). Little data regarding *HOX* gene expression are available for the bovine embryo around gastrulation or thereafter [15–17]. However, a transcriptomic study revealed *HOXB9* expression in bovine embryos from day 7 to day 19 post-insemination (D7 to D19—[17]). Moreover, data concerning abundance and expression of HOX proteins are largely lacking for the majority of developmental stages in most mammalian species.

Although HOX proteins are best known for their roles in the context of embryo shaping in relationship with gastrulation, several *HOX* transcripts have been detected quite earlier during development in a number of mammalian species [18–27]. In particular, we have previously shown that *HOXB9* transcripts are present in oocytes and early embryos in the mouse and bovine [24]. In the bovine, the relative expression of *HOXB9* increases between the immature oocyte and the zygote stage, further increases at the 5- to 8-cell stage and peaks at the morula stage before decreasing at the blastocyst stage. In the mouse, *Hoxb9* transcripts are also detected at all those early developmental stages. Zygotic and maternal HOXB9 does not appear to be crucial for oocyte/embryo development since *Hoxb9* ^-/-^ homozygous mice are obtained with the expected Mendelian frequency and mutant animals of both sexes are viable and fertile [8]. However, so far no study has precisely investigated the possible impact of HOXB9 loss of function on the first embryonic stages. Moreover, the significant changes in relative transcript levels observed during early development suggest a specific regulation of *HOXB9*. This may be linked to specific role(s) of the protein during mammalian oocyte maturation and early embryogenesis, such as in the control of the major embryonic genome activation (EGA—[28]) or the first cell differentiation event leading to inner cell mass (ICM) and trophectoderm (TE) formation.

In the present study, we have characterized the distribution of the protein in mouse and bovine oocytes and early embryos up to the peri-gastrulation period, for the first time. To this end, specific antibodies directed to HOXB9 were carefully selected and tested for each species. Protein presence and localization were then evaluated by whole-mount immunofluorescence or immunohistochemistry at several developmental stages. Protein markers were simultaneously used to precisely localize HOXB9 in specific cell lineages or subcellular compartments. The two mammalian models were chosen due to their differences in kinetics of development, timing of EGA, implantation (E4.5 mouse embryo is ready to implant, while bovine embryo elongates and is free in the uterus until around D21) or extra-embryonic tissue development (yolk sac wall and amnion composition as well as allantois development—[29–32]).

## Materials and Methods

Chemicals were purchased from Sigma-Aldrich (Saint-Louis, USA) unless otherwise stated.

### Embryo production

#### *In vitro production* of bovine embryos

Bovine embryos were produced, as previously described [24]. In brief, bovine ovaries were collected at a local slaughterhouse. Cumulus-oocyte complexes (COCs) were aspirated from 3–8 mm follicles, selected and washed three times in Hepes-buffered Tissue Culture Medium 199 (TCM-199). Groups of 80 to 100 COCs were matured for 24 h at 39°C under 5% CO_2_ in air in 500 μl of enriched serum-free maturation medium [33]. Frozen bull semen was kindly provided by the Association wallonne de l’Elevage (Ciney, Belgium). After thawing, living spermatozoa were isolated on a discontinuous Percoll gradient and then co-incubated with matured COCs at a final titer of 2 x 10^6^/ml for 18 h in a modified Tyrode’s albumin lactate pyruvate medium supplemented with 6 mg/ml fatty acid-free fraction V BSA and 1.7 IU/ml heparin. The temperature and gas composition were the same as described for the maturation step. After fertilization, presumptive zygotes were denuded by vortexing and transferred by groups of 25 to 30 into culture droplets covered with mineral oil (FertiCult^™^, Fertipro, Beernem, Belgium). Culture medium consisted of modified Synthetic Oviduct Fluid [34] supplemented with BSA (4 mg/ml) and ITS (5 μg/ml of insulin, 5 μg/ml of transferrin and 5 ng/ml of selenium—[35]). Embryos were cultured at 39°C under 5% O_2_, 5% CO_2_ and 90% N_2_.

Oocytes were harvested before and after maturation. One-cell embryos, 2-cell embryos, 5- to 8-cell embryos, 9- to 16-cell embryos and compact morulae were collected 18 h post-insemination (hpi), 26 hpi, 48 hpi, 96 hpi and 120 hpi, respectively, while blastocysts were obtained at day 6, 7, 7.5, 8, 8.5 and 9 post-insemination (D6, D7, D7.5, D8, D8.5 and D9).

#### Collection of *in vivo* bovine embryos

*In vivo* D7 embryos were a gift from the Association wallonne de l’Elevage (Belgium). D7 embryos were received cryopreserved and were thawed as previously described [36] with slight modifications.

After oestrus synchronization and ovarian stimulation, heifers were inseminated (day 0) and elongating bovine embryos from D11 to D18 were harvested by non-surgical flushing of the uterus [37], while D25 embryos and allantoises were collected by uterine flushing after uterus dissection at a slaughterhouse [38]. Animal use and care were performed in accordance with the International Guiding Principles for Biomedical Research involving Animals at the INRA experimental farm (registered under N° FRTB910 in the national registry) and the protocol for this study was approved by the local Ethics Committee (Comité d’Ethique en Expérimentation Animale du Centre INRA de Jouy-en-Josas et AgroParisTech [or COMETHEA], registered as 12/084 and 12/086 in the National Ethics Committee registry).

#### Mouse oocyte collection and embryo culture

Investigations on mice were approved by the animal ethics committee of the Université catholique de Louvain (approval 083001) and were in accordance with the European directive 2010/63/UE. For mouse euthanasia, CO_2_ was administered by progressive delivery in the cage volume in accordance with European guidelines. All experiments were carried out on F1 hybrid mice: DBA/2N x C57BL/6J (Charles River Laboratories, Brussels, Belgium).

Female mice (8- to 9-week-old) were superovulated, as previously described [24]. Immature oocytes were harvested 47 h post-eCG whereas mature oocytes were collected 14 h post-hCG injection. Superovulated mice were mated overnight (O/N) and euthanized 21 h post-hCG to collect presumptive zygotes from the oviductal ampulla. Zygotes were then denuded from their cumulus cells and cultured, as previously described [24]. Two-cell embryos, pre-compaction morulae, compact morulae, early blastocysts and blastocysts were collected 48 h (E1.5), 72 h (E2.5), 80 h (E2.8), 92 h (E3.3) and 100 h (E3.6) post-hCG injection, respectively.

#### Collection of *in vivo* mouse embryos

Collection of in vivo mouse embryos. Post-implantation embryos were produced *in vivo* and harvested after euthanasia at E4.5 by uterus flushing, and at E5.5, E6.5, E7.5 (late allantoic bud [LB] and early headfold—late headfold [EHF—LHF] stages), E7.8 (first somites stage) and E12.5 by uterus dissection. For E6.5 and later stages, Reichert’s membrane was removed from the embryo to facilitate penetration of antibodies. *Hoxb9*^-/-^ E12.5 embryos were kindly provided by D. Wellik (University of Michigan, Ann Arbor, USA—[8]).

### Cell culture

HEK293T cells (ATCC CRL-3216^™^, Molsheim, France) were cultured at 37°C under 5% CO_2_ in DMEM gluta Max^™^-II (Gibco, Gent, Belgium) supplemented with 10% fetal bovine serum (FBS—Gibco), 100 U/ml Penicillin (Gibco), 100 μg/ml Streptomycin (Gibco) and 1 mM Na-pyruvate (Gibco).

### Hox9 plasmid construction

pUC57 plasmid containing the coding region for bovine *HOXA9* (NM_001105617.2), *B9* (XM_001251856, new update on 30^th^ December 2014: NM_001191186.1), *C9* (XM_002687231.2, new update on 30^th^ December 2014: XM_002687231.4) or *D9* (XM_002685288.1, new update on 30^th^ December 2014: XM_002685288.3) were purchased from GenScript (Piscataway, USA). The mouse *Hox9* expression vectors were kindly provided by D. Wellik (University of Michigan, Ann Arbor, USA). Using the Gateway^®^ system, sequences of interest were transferred into a v1899 destination vector for the expression of proteins fused to a N-terminal triple-FLAG tag [39]. pCAT^®^3-vector (Genbank: U57025) was obtained from Promega (Madison, USA).

### Immunostaining and imaging

Primary and secondary used antibodies are listed in Tables 1 and 2, respectively. A polyclonal anti-human HOXB9 antibody (n°1) directed against a sequence showing 100% identity with the bovine protein was selected to highlight bovine HOXB9 protein, while a polyclonal anti-human HOXB9 antibody (n°2) directed against a sequence showing 98% identity with the mouse protein was selected to detect HOXB9 in the mouse. Throughout the figures, HOXB9 staining appears in red, whatever the used fluorochromes, in order to facilitate the reading of the paper.

**Table 1:** Primary antibodies used for proteins detection.

Antibodies	Species	Clonality	Reference	Application
**BioGenex**				
CDX2^1^	Mouse	Monoclonal	#MU392A-UC^a^	IH, IF: 1/100
CDX2^2^	Mouse	Monoclonal	#AM392-5M^b^	IF: N/A
**Santa Cruz Biotechnology**				
DLX3^3^^,^ ^4^	Goat	Polyclonal	#Sc-18143	IF: 1/100
GATA4 (C-20)^5^^,^^6^	Goat	Polyclonal	#Sc-1237	IF: 1/50
HOXB9 (H-80, N°2)	Rabbit	Polyclonal	#Sc-66924	IC, IH, IF: 1/100 WB: 1/200
OCT3/4 (N-19)^5^	Goat	Polyclonal	#Sc-8628	IF: 1/50
Vimentin (V9)^4^	Mouse	Monoclonal	#Sc-6260	IF: 1/100
**Sigma-Aldrich**				
β-actin HRP	Mouse	Monoclonal	#A3854	WB: 1/20.000
FLAG	Mouse	Monoclonal	#F1804	IC: 1/100 WB: 1/5000
HOXB9 (N°1)	Rabbit	Polyclonal	#AV32639	IC, IH: 1/300 IF: 1/500 WB: 1/1000
**R&D Systems**				
Brachyury^7^	Goat	Polyclonal	#AF2085	IF: 1/100
CER-1^7^	Rat	Monoclonal	#MAB1986	IF: 1/150
GATA6	Goat	Polyclonal	#AF1700	IF: 1/200

IF: whole-mount immunofluorescence, IH: immunohistochemistry, IC: immunocytochemistry, WB: western-blot. Antibody used for post-implantation ^a^ or pre-implantation ^b^ embryos.

^1^ Blij et al. [40];

^2^ Goossens et al. [41];

^3^ Degrelle et al. [42];

^4^ Hue et al. [16];

^5^ Papanayotou et al. [43];

^6^ Plusa et al. [44];

^7^ Hoshino et al. [45].

**Table 2:** Secondary antibodies used for proteins detection.

Antibodies	Species	Reference	Application
**Cell Signaling**			
Anti-rabbit Alexa Fluor^®^555	Goat	#4413	IC, IH: 1/500 IF: 1/500 and 1/1000
Anti-mouse Alexa Fluor^®^488	Goat	#4408	IC, IH, IF: 1/500
**Santa Cruz Biotechnology**			
Anti-rabbit HRP	Bovine	#Sc-2370	WB: 1/10.000
Anti-mouse HRP	Goat	#Sc-2005	WB: 1/10.000
**Thermo Fisher Scientific**			
Anti-goat Alexa Fluor^®^555	Donkey	#A-21432	IF: 1/500
Anti-goat Alexa Fluor^®^633	Donkey	#A-21082	IF: 1/400
Anti-goat Alexa Fluor^®^647	Donkey	#A-21447	IF: 1/200
Anti-mouse Alexa Fluor^®^488	Donkey	#A-21202	IF: 1/400
Anti-rabbit Alexa Fluor^®^488	Donkey	#R37118	IF: 1/500
Anti-rabbit Alexa Fluor^®^546	Donkey	#A10040	IF: 1/200

IF: whole-mount immunofluorescence, IH: immunohistochemistry, IC: immunocytochemistry, WB: western-blot.

For each immunostaining performed in this study, a negative control without primary antibodies was involved.

#### Whole-mount immunofluorescence

Bovine and mouse whole-mount immunofluorescence was performed on oocytes/embryos until the blastocyst stage (bovine: until D11, mouse: until E3.5), as previously described with slight modifications [46]. In brief, oocytes/embryos were fixed in 2% paraformaldehyde (PFA) in phosphate buffered saline (PBS). A working solution of 0.5% (WS 0.5) or 0.1% (WS 0.1) PBS-Tween20 was used for the bovine and mouse, respectively. Samples were permeabilized with 0.5% Triton X-100 in WS. They were then blocked in 10% BSA in WS, followed by incubation in primary solution (1% BSA in WS). After incubation with the primary antibody, samples were washed in WS and incubated with secondary antibody before being rinsed again. Embryos were mounted in Vectashield^®^ containing DAPI (Vector Laboratories, Burlingame, USA) on Lab-Tek^®^ chamber slides (#155411, Thermo Fisher Scientific, St Leon-Rot, Germany).

Mouse post-implantation (from E4.5 to E7.5) embryos were fixed in 4% PFA in PBS and were permeabilized with 1% Triton X-100 in 0.5% Tween20 in PBS. After blocking in 10% BSA in 0.1% Triton X-100 in PBS, they were incubated with the primary antibody diluted in PBS containing 1% BSA and 0.1% Triton X-100. Embryos were rinsed and incubated in secondary antibody solution. Finally, they were washed. All the rinsing and incubation steps were performed on an agitator plate. Embryos were mounted in Vectashield^®^ containing DAPI in a circle of nail-polish drew on a slide to avoid crushing the embryo.

Temperature and incubation time of the whole-mount immunofluorescence steps are summarized in Table 3 according to the biological samples.

**Table 3:** Conditions of whole-mount immunofluorescence on bovine and mouse embryos.

	Oocytes/Embryos until blastocyst stage		Mouse post-implantation stages			
	Bovine	Mouse	E4.5	E5.5	E6.5	E7.5
**Fixation (RT)**	1 h	20 min	30 min	30 min	30 min	30 min
**Permeabilization (RT)**	1 h	1 h	1 h	1 h	1 h	2 h
**Blocking**	1 h; RT	1 h; RT	1 h; RT	1 h; RT	O/N; 4°C	O/N; 4°C
**ACI incubation (4°C)**	O/N	O/N	O/N	O/N	O/N	O/N
**Rinsing (RT)**	3 x 5 min	3 x 5 min	3 x 5 min	3 x 20 min	3 x 60 min	3 x 60 min
**ACII incubation**	1 h; RT	1 h; RT	1 h; RT	O/N; 4°C	O/N; 4°C	O/N; 4°C
**Rinsing (RT)**	3 x 5 min	3 x 5 min	3 x 5 min	3 x 20 min	3 x 60 min	3 x 60 min

ACI: primary antibody, ACII: secondary antibody, RT: room temperature, O/N: overnight.

#### Immunohistochemical and immunocytochemical analysis

Bovine elongating conceptuses (D14 and D17) and allantois were fixed from 3 h to O/N in 4% PFA in PBS and incubated successively in 15% and 18% sucrose in PBS before being embedded in Tissue-Tek^®^. Serial transversal sections of 10 μm were cut with a Leica CM 3050S cryostat (Leica Biosystems, Diegem, Belgium).

E12.5 mouse embryos were washed in PBS and fixed in 4% PFA in PBS for 1 h at 4°C. Fixed embryos were then rinsed three times for 5 min in 1% PBS-Tween20 and dehydrated as followed: 5 min in a 25% MetOH, 5 min in a 50% MetOH, 5 min in a 75% MetOH and 2 times 5 min in a 100% MetOH. An inverted MetOH gradient was used to rehydrate the embryos. After O/N incubation in 30% sucrose in PBS solution, cryopreserved embryos were embedded in Shandon^™^ Cryomatrix^™^ (Thermo Scientific). Frozen 18 μm sections were rehydrated in PBS for 5 min.

HEK293T cells were seeded on glass coverslips coated with poly-D-lysine (0.01 mg/ml) and transfected with 500 ng of the corresponding plasmid using the jetPrime^™^ system (Polyplus transfection^™^, Illkirch, France), following the manufacturer’s instructions. Cells were then fixed for 20 min in 4% PFA in PBS.

For immunochemistry, sections or cells were permeabilized with 0.5% Triton X-100 in WS 0.1 for 10 min at RT and blocked in 10% skimmed powder milk in WS 0.1 for 1 h at RT. Samples were then successively incubated in primary (O/N at 4°C) and secondary (1 h at RT) antibody solutions (1% BSA in WS 0.1). Antibody incubations were followed by three rinsing steps of 5 min in WS 0.1. Samples were mounted in Vectashield^®^ containing DAPI.

#### Combined whole-mount immunofluorescence and immunohistochemical analysis

D15 and D18 bovine embryos were successively submitted to a whole-mount immunofluorescence and to an immunohistochemical analysis on slices obtained after cutting the stained embryos. Briefly, embryos were fixed, dehydrated and stored at -20°C until used, as previously described [16]. Embryos were then rehydrated using a decreasing methanol gradient: 75%, 50%, and 25% for 15 min each and for 1 h in PBS. After being permeabilized in 0.5% triton in WS 0.1 for 40 min at RT, embryos were blocked with a 5% BSA IgGs free solution in WS 0.1 for 1 h at RT. Then, embryos were incubated O/N at 4°C in the primary antibody solution containing 5% BSA in WS 0.1. After rinsing, embryos were incubated with the secondary antibody solution for 1 h at RT and rinsed again before being incubated in a DAPI solution (1/20.000) for 10 min at RT. Embryos were rinsed and mounted in Fluoromount-G^®^. After images acquisition, the embryos were recovered and rinsed (for 2 h at RT in WS 0.1) and re-fixed in 4% PFA in PBS for 10 min at RT. Embryos were then embedded in Tissue-Tek^®^ and 5 μm sections were obtained. Slices were dried for 15 min at RT, incubated for 10 min in PBS and permeabilized for 30 min at RT in the same permeabilization solution used for the whole-mount immunofluorescence. Embryos were then blocked and successively incubated in the primary and secondary antibody solutions, as described previously. Slices were mounted with Dako fluorescent mounting medium (Dako, Les Ulis, France).

#### Imaging

The labeled oocytes, embryos and cells were observed under a confocal microscope using the ZEN acquisition software (LSM710, Zeiss, Jena, Germany) or with an Axioskop 2 microscope. Images of D15 and D18 bovine embryos were acquired using a Nikon AZ100 multizoom microscope (Nikon, Amsterdam, The Netherlands) for the whole-mount or using a Lamina multilabel slide scanner (Perkin Elmer, Massachusetts, USA) for sections.

Blastocyst fluorescence signal was quantified using Nis-element 3.1 (Nikon) software. For each ICM or TE cell, the mean fluorescence intensity (i.e. mean intensity of pixels) of the nucleus was quantified and reported to the geometric mean of mean intensity of all the ICM nuclei taken as reference.

### Western-blot

HEK293T cells seeded in 6-well plates were transfected with 1 μg of expression vector using the jetPrime^®^ reagent following the instructions of the manufacturer. Proteins were extracted with IPLS buffer (20 mM Tris HCl pH 7.5, 120 mM NaCl, 0.5 mM EDTA, 0.5% Nonidet P40, protease inhibitor [#11873580001, Roche, Mannheim, Germany], 10% glycerol). After 5 min boiling, protein extracts were run on 10% SDS-PAGE gels and blotted on nitrocellulose membranes (#10600002, Amersham Biosciences, Diegem, Belgium). Membranes were blocked for 1 h at RT in 10% skimmed powder milk and were then successively incubated at 4°C with primary (O/N) and secondary (1 h) antibody solutions. Primary and HRP-conjugated secondary antibodies used are listed in Tables 1 and 2. Peroxidase activity was detected using the western lightning^®^ Plus-ECL system (#NEL104001EA, PerkinElmer, Massachusetts, USA). Membranes were recycled by stripping (5 min in water, three times for 5 min in 0.2 M NaOH and 5 min in water) and blocked again for protein load detection using β-actin HRP (1 h at 4°C).

#### Statistical analysis

Statistical analyses were performed with JUMP Pro 12 software (SAS Institute, Cary, USA). The HOXB9 distribution between mouse or bovine ICM and TE cells was analyzed at each stage by comparing the ratios obtained for all nuclei with a linear mixed model. The cell lineage (ICM or TE) was considered as fixed variable, while the embryo and the experiment were considered as random variables. Interactions between embryo and experiment factors have also been analyzed using REML variance component estimates. Differences were considered significant at p-values lower than 0.05. Ratios of ICM or TE cells were compared between stages with a Welch test. For significant differences, each pair was then compared with a Student T test for unequal variances. The significant threshold was adjusted following the Bonferroni method: a p-value below 0.05/3 (0.017) was significant.

## Results

### Validating antibodies for the bovine and mouse HOXB9 proteins

**Fig 1:**
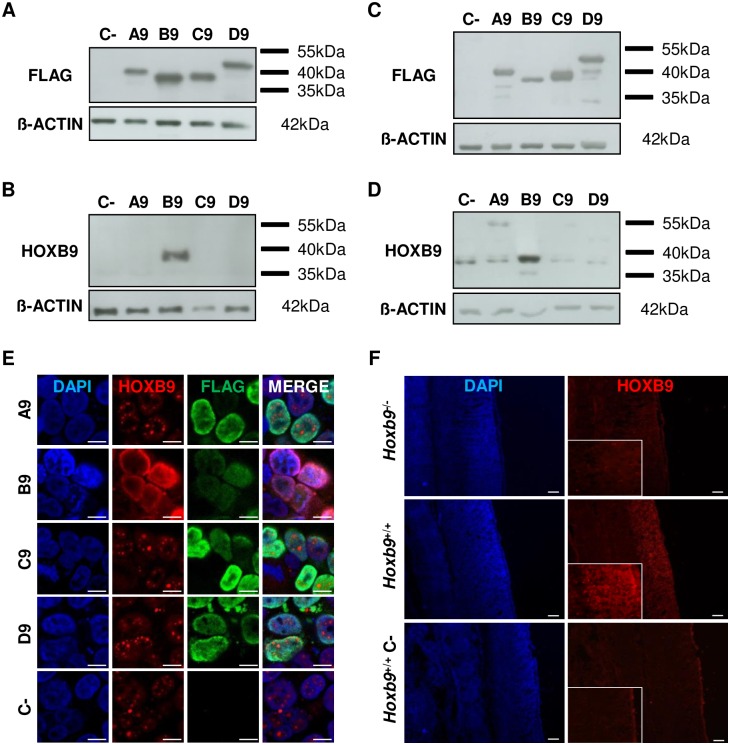
Analysis of specificity of the anti-HOXB9 antibodies. **A-E:** Overexpression of mouse **(A-B)** or bovine **(C-E)** HOXA9 (A9), HOXB9 (B9), HOXC9 (C9) or HOXD9 (D9) proteins in HEK293T cells. Each protein was fused to a FLAG tag. **(A, C)** Western-blot using the anti-FLAG antibody (N = 3). **(B, D)** Western-blot using the anti-HOXB9 antibody 1 **(D)** or 2 **(B)** (N = 3). C-: cells transfected with a control vector. The ß-ACTIN was used to control protein loading. **(E)** HEK293T cells overexpressing the bovine HOX9 proteins or transfected with a control vector (C-) were stained simultaneously with anti-HOXB9 (Red) and anti-FLAG (Green) antibodies and DAPI (N = 3). Representative confocal Z-section. Scale bar = 10 μm. **F:** Immunofluorescence with anti-HOXB9 antibody 2 on E12.5 *Hoxb9*^-/-^ knock-out mouse embryos (N = 2). E12.5 wild-type embryos (*Hoxb9*^+/+^) were used as positive control. *Hoxb9*^+/+^ C-: negative control without primary antibody. The boxes represent zooms on neural tube epithelium. HOXB9: Red, DAPI: Blue. Epifluorescence images. Scale bar = 100 μm. N = number of replicates.

The specificity of the two antibodies was assayed in HEK293T cells overexpressing bovine or mouse HOXB9 protein, respectively, via two complementary approaches: western-blot detection on protein extracts (N = 3 –Fig 1A–1D) and immunofluorescence (N = 3 –Fig 1E). Moreover, to ensure that the antibodies do not recognize other HOX proteins of the paralogous group 9 (due to high sequence homology between HOX9 proteins), cells overexpressing HOXA9, HOXC9 or HOXD9 were also tested.

The western-blot performed with the anti-FLAG antibody revealed a robust expression of mouse or bovine HOX9 proteins (Fig 1A and 1C). Spots associated to the proteins were observed at molecular weights appearing slightly higher than the theoretical ones (HOXA9: ± 30 kDa, HOXB9: ± 28 kDa, HOXC9: ± 29 kDa and HOXD9: ± 35 kDa) probably due to the denaturating conditions used. Using the anti-HOXB9 antibodies, one band of high intensity was observed for both species only in cells transfected for the corresponding HOXB9 expression constructs and not for the other HOX9 paralogues (Fig 1B and 1D). This band appears at the same molecular weight as observed with the anti-FLAG antibody. This demonstrates that the selected antibodies do not cross-react with paralogous proteins thereby supporting their specificity towards HOXB9. A band corresponding to the HOXB9 protein was also detected with a lower intensity in controls (Fig 1D), confirming a basal level of expression of *HOXB9* in HEK293T cells [47]. This was further confirmed by RT-PCR. Antibody specificity was also checked by immunofluorescence for the bovine (Fig 1E) and mouse proteins. A double staining was performed with anti-HOXB9 antibody and an anti-FLAG antibody. A strong nuclear staining corresponding to FLAG-tagged HOXB9 proteins was observed with the anti-HOXB9 antibody while only the FLAG epitope was detected in FLAG-tagged HOXA9, HOXC9 or HOXD9 expressing cells.

Immunohistochemistry was also performed on E12.5 *Hoxb9*^*-/-*^ embryos to confirm the specificity of the antibody in the mouse (N = 2). No signal was observed in *Hoxb9*^*-/-*^ embryos, while in wild-type embryos HOXB9 proteins were detected in the dorsal part of the epithelium of the neural tube (Fig 1F) and with an anterior limit corresponding to the one described for *Hoxb9* transcripts [8, 48, 49].

Together, these data confirm the specificity of both HOXB9 antibodies.

### HOXB9 is present in oocytes and from the zygote to the blastocyst stage in mammals

To correlate the presence of HOXB9 proteins with *HOXB9* expression profiles previously revealed in the mouse and bovine [24], distribution of proteins was characterized using whole-mount immunofluorescence in oocytes and from the zygote to the blastocyst stage. For each stage, at least 3 embryos were handled at the same time and experiments were repeated at least twice.

#### Mouse

**Fig 2:**
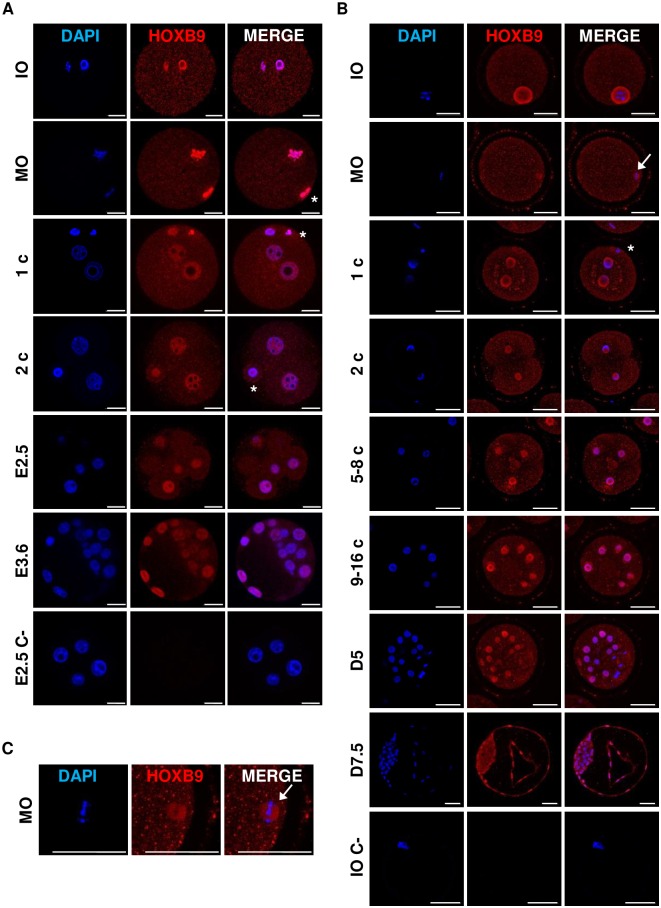
Mouse and bovine HOXB9 protein distribution in oocytes and from zygotes to blastocysts. **A-C:** Whole-mount immunofluorescence of *in vitro* cultured mouse **(A)** and *in vitro* produced bovine **(B)** embryos. Immature oocyte (IO); mature oocyte (MO); 1-cell embryo (1c); 2-cell embryo (2 c); 5- to 8-cell embryo (5–8 c); 9- to 16-cell embryo (9–16 c); mouse pre-compaction stage (E2.5); bovine compact morula (D5); mouse blastocyst (E3.6) and bovine blastocyst (D7.5). Negative control without primary antibody are shown for E2.5 mouse embryos (E2.5 C-) and bovine IO (IO C-). **(C)** Zoom on bovine HOXB9 distribution at the metaphasic plate of mature oocyte. Nuclei: Blue; HOXB9: Red. The asterisk depicts polar body. White arrow shows metaphasic plate. Representative confocal Z-section. Scale bar = 20 μm (mouse) or 50 μm (bovine).

Mouse HOXB9 immunofluorescence was detected at all analyzed stages (Fig 2A). In mouse immature and mature oocytes, a strong HOXB9 staining overlapped with the DAPI, while a fainter cytoplasmic staining was also present. A strong signal was detected in the maternal and the paternal pronuclei of zygotes as well as in polar bodies (asterisk), whereas a lighter signal was present in the cytoplasm. In 2-cell embryos and pre-compaction morulae (E2.5), HOXB9 was found in the nucleus and cytoplasm of all cells. The nuclear staining was always more prominent than the cytoplasmic one at these stages. At the blastocyst stage (E3.6), heterogeneity of HOXB9 staining was observed between ICM and TE cells, with a stronger staining in the nucleus of both polar and mural TE cells.

#### Bovine

As in the mouse, HOXB9 was present in bovine oocytes and throughout early embryonic development (Fig 2B). In immature oocytes, a staining was visible in the cytoplasm but the germinal vesicle was much more intensely stained. HOXB9 did not co-localize with the DAPI staining of immature and mature oocytes, conversely to the mouse. Also, in mature oocytes, HOXB9 staining was slightly more intense at the metaphasic plate for 24 out of the 28 mature oocytes analyzed (Fig 2B and 2C—arrow). From the zygote to the blastocyst stage, the presence of HOXB9 was more visible in the nuclei than in the cytoplasm, similarly to the mouse. A strong signal was detected in the maternal and the paternal pronuclei of zygotes. The polar bodies were also stained. In D7.5 blastocysts, as observed for the mouse, HOXB9 was present in both cell lineages with a stronger nuclear staining in TE cells.

These results show that mouse and bovine HOXB9 protein profiles are similar and characterized by the presence of the protein in all oocytes and embryonic cells from the zygote to the blastocyst stage, mainly in nuclei.

### The HOXB9 protein is expressed differently in the two first cell lineages of mammalian blastocysts

An in-depth study of HOXB9 protein distribution in ICM and TE cells was conducted on *in vitro* cultured mouse compact morulae (E2.8) and blastocysts (E3.3 and E3.6) and on *in vitro* produced bovine embryos from early to hatched blastocysts (D6, D7.5 and D8). At least 11 mouse embryos from 2 replicates and 14 bovine embryos from 3 repetitions were analyzed at each stage. Mouse and bovine embryo development from fertilization to gastrulation is reviewed elsewhere [31, 50–57].

#### Mouse

**Fig 3:**
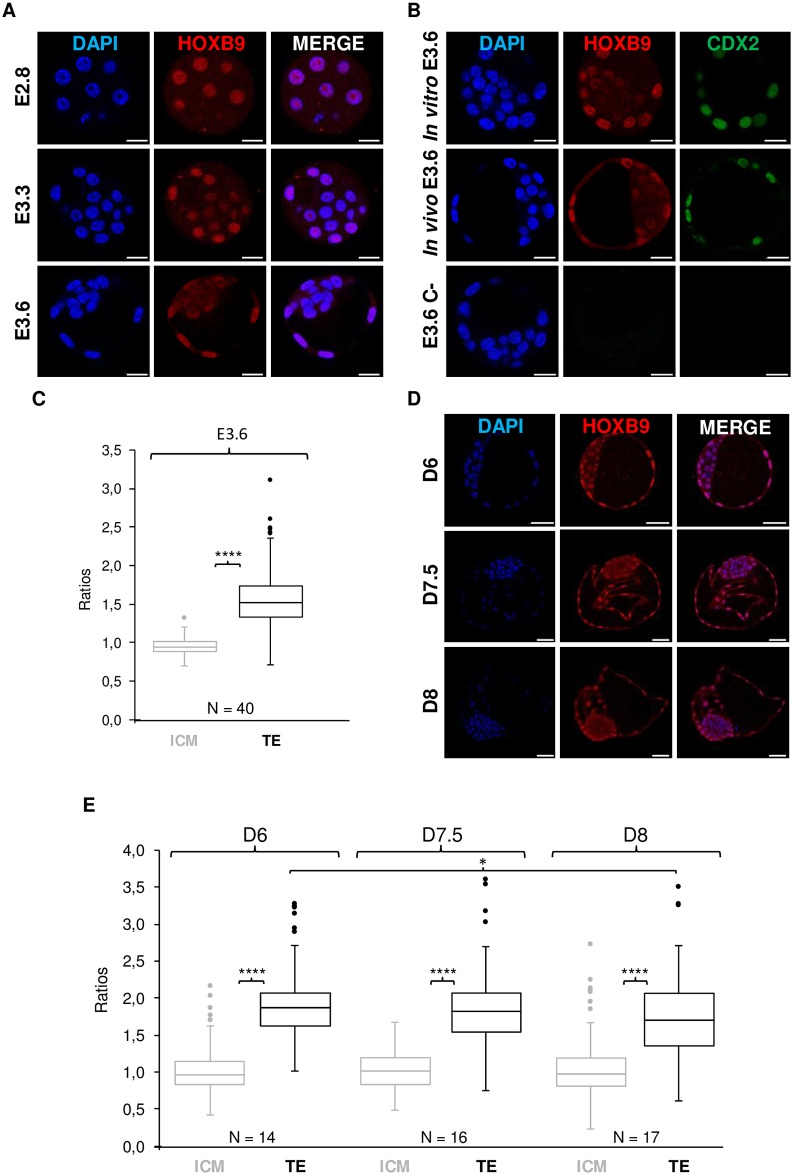
In-depth study of mouse and bovine HOXB9 protein distribution during the first step of cell differentiation. **A-C:** Mouse HOXB9 protein distribution. **D-E:** Bovine HOXB9 protein distribution. **(A-B, D)** Whole-mount immunofluorescence. Mouse compact morula at embryonic day E2.8 (E2.8); mouse blastocyst at E3.3 and E3.6 and bovine blastocyst at day 6, 7.5 and 8 post-insemination (D6, D7.5 and D8). Nuclei: Blue; HOXB9: Red; CDX2: Green. Representative confocal Z-section. Scale bar = 20 μm (mouse) or 50 μm (bovine). **(C, E)** Quantification of relative fluorescence corresponding to nuclear HOXB9 proteins. The boxplot depicts the distribution of the ratios. The ends of the whiskers represent the lowest or the highest datum still within 1.5 x interquartile range. Dots correspond to outliers. N = number of analyzed embryos. * Significant difference (Linear mixed model, * = 0.01 < p < 0.05, **** = p < 0.0001).

All nuclei of compact morulae presented a similar staining, similarly to that previously described at the pre-compaction stage. Conversely, in E3.3 and E3.6 blastocysts, the nuclei of both polar and mural TE cells displayed increased staining compared to ICM cells nuclei (Fig 3A). A similar pattern was observed in *in vivo* produced blastocysts (Fig 3B). To confirm this observation, nuclear staining intensity was quantified and compared between ICM and TE cells. To accurately identify cells belonging to one or the other cell lineages, a co-staining with CDX2, a transcription factor specific to TE cells [58, 59], was performed (Fig 3B). Forty embryos from 3 repetitions were analyzed (total number of analyzed nuclei: ICM = 217, TE = 258). Relative fluorescence was significantly higher for TE cells than for ICM cells (linear mixed model, p < 0.0001—Fig 3C) suggesting that HOXB9 is more abundant in TE than in ICM nuclei. Furthermore, a majority of E3.6 blastocysts displayed heterogeneity between ICM cells for HOXB9 nuclear staining which was not observed in E3.3 blastocysts.

#### Bovine

As observed for the mouse, while all cells of compacted morulae displayed a similar nuclear staining (Fig 2B), TE nuclei displayed strong staining compared to ICM at all the blastocyst stages (Fig 3D). Quantification of the staining confirmed this observation. Indeed, relative staining was significantly higher in TE cells than in ICM cells at all the blastocyst stages (linear mixed model, p < 0.0001—Fig 3E). Fourteen D6 blastocysts (number of analyzed nuclei: ICM = 190 and TE = 135), 16 D7.5 blastocysts (number of analyzed nuclei: ICM = 250 and TE = 205) and 17 D8 blastocysts (number of analyzed nuclei: ICM = 264 and TE = 227) were analyzed. In addition, in line with observations made in E3.6 mouse blastocysts, heterogeneity of HOXB9 staining among ICM nuclei was clear from the D7.5 blastocyst stage (Fig 3D).

These results demonstrate that mouse and bovine HOXB9 nuclear proteins are equally distributed between cells in the embryo until compaction, before being reduced in ICM cells compared to TE cells. Furthermore, HOXB9 abundance in the nucleus become heterogeneous from cell to cell in ICM during blastocyst expansion.

### The nuclear abundance of HOXB9 heterogeneously decreases in ICM during primitive endoderm formation

The final event in blastocyst formation is the segregation of ICM cells into the primitive endoderm (PrE) and epiblast. Precursors of these two cell lineages are present and display a salt and pepper distribution in ICM prior their final commitment (taking place at E4.5 for the mouse and at D12 for the bovine). Both of these cell types express specific markers [55, 60–62]. We hypothesized that the heterogeneity of HOXB9 staining observed between ICM cells of E3.6 mouse and D7.5-D8 bovine blastocysts could be related to the segregation of PrE and epiblast cells. To test this hypothesis, specific markers of PrE cells, namely GATA4 in the mouse [43, 44, 55, 63] and GATA6 in the bovine [61, 62], were detected by immunofluorescence. Mouse epiblast cell nuclei were also co-stained with OCT4 [43, 63, 64].

#### Mouse

**Fig 4:**
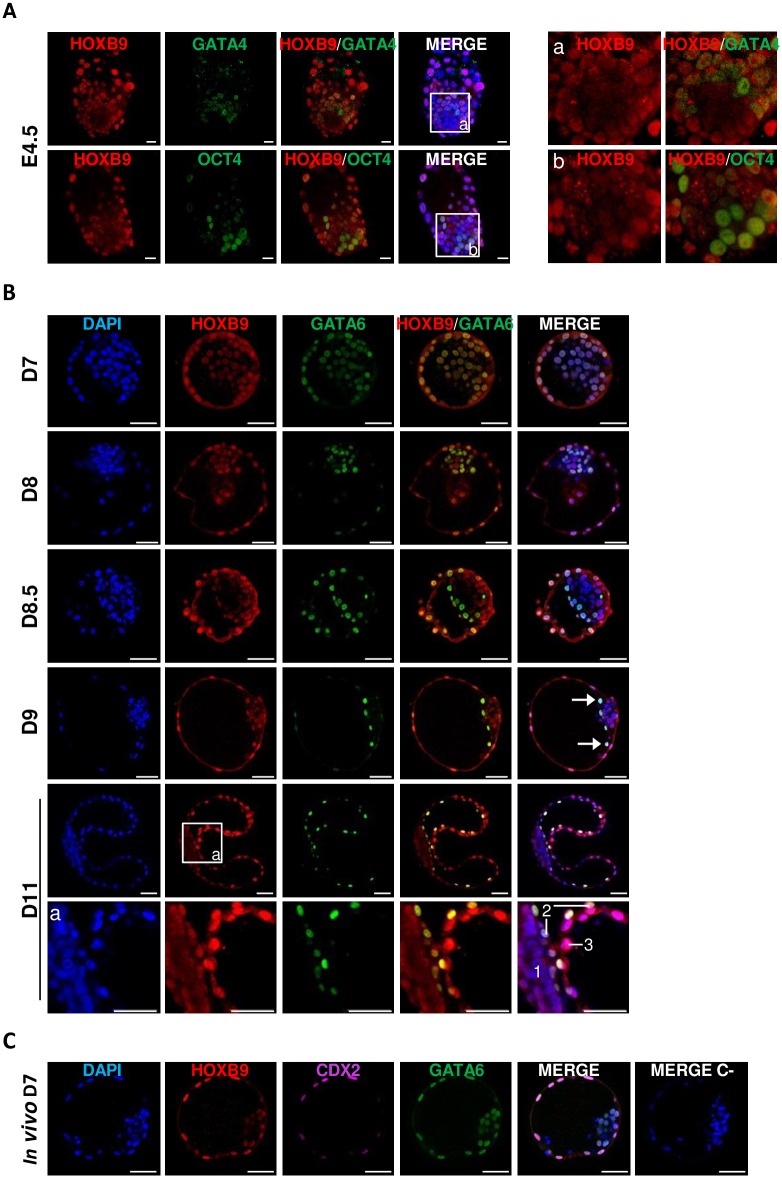
Mouse and bovine HOXB9 protein distribution during primitive endoderm formation. **A-C:** Whole-mount immunofluorescence. **(A)** Mouse *in vivo* blastocyst at E4.5. a—b: Zoom on epiblast. **(B)** Bovine *in vitro* blastocysts at day 7, 8, 8.5 and 9 post-insemination (D7; D8; D8.5 and D9) and *in vivo* embryos at day 11 post-insemination (D11). Arrows point to GATA6 positive cells that could correspond to primitive endoderm cells. Zoom on D11 bovine embryo **(a)**. 1. epiblast; 2. primitive endoderm; 3. trophectoderm. **(C)** Bovine blastocyst produced *in vivo* at D7. Merge C-: negative control without primary antibody. Nuclei: Blue; HOXB9: Red; GATA4/GATA6: Green; CDX2: Pink. Representative confocal Z-section. Scale bar = 20 μm (mouse) or 50 μm (bovine).

E4.5 mouse embryos produced *in vivo* from 3 replicates (8 embryos) were analyzed. As observed in E3.6 blastocysts, all mural and polar TE cells presented a stronger nuclear HOXB9 staining than ICM cells, in which HOXB9 showed different level of expression between cells (Fig 4A). No correlation between HOXB9 and GATA4 staining could be established since GATA4 positive or negative nuclei displayed variable HOXB9 staining. Similarly, no correlation was found between OCT4 and HOXB9 staining intensities (3 replicates, 8 embryos—Fig 4A).

#### Bovine

*In vitro* produced blastocysts were collected from 3 replicates at D7, D8, D8.5 and D9. Moreover, *in vivo* D7 and D11 embryos were also analyzed. To verify if a similar HOXB9 pattern was observed in *in vitro* and *in vivo* embryos, HOXB9 staining was furthermore analyzed in *in vivo* produced D7 blastocysts. From D8 on, all collected blastocysts were hatched. As previously observed, TE nuclei showed stronger staining associated to HOXB9 than ICM nuclei at all analyzed stages (Fig 4B). At D7, all nuclei were positive for GATA6 and HOXB9. Moreover, half of the embryos showed heterogeneity in ICM staining for HOXB9. *In vivo* produced D7 blastocysts presented the same pattern (Fig 4C), suggesting the absence of impact of culture conditions on *HOXB9* expression. In D8 hatched blastocysts, GATA6 expression displayed a “salt and pepper” pattern. All cells remained HOXB9 positive and 4 out of 6 embryos showed heterogeneity in ICM staining for HOXB9 from cell to cell nuclei. From D8.5 to D9, the majority of GATA6 positive cells were localized at the limit between ICM and the blastocoel cavity. These cells likely correspond to PrE cells. Most of them displayed a clear nuclear HOXB9 staining. In some embryos, isolated cells localized against TE were present and could also correspond to PrE cells (Fig 4B—arrows). Those cells displayed a clear HOXB9 staining. Moreover, negative GATA6 nuclei showed various intensity of HOXB9 staining. This pattern was previously observed in a small number of D8 blastocysts. At D11, PrE cells showed HOXB9 nuclear staining. It was also interesting to notice that the HOXB9 nuclear staining was still reduced in epiblast cells but the staining was more homogeneous between cells unlike what was observed in ICM of D7.5–9 embryos.

To conclude, neither mouse nor bovine nuclear HOXB9 proteins abundance correlates with the presence of PrE markers. However, in the bovine, once PrE formation is initiated, all corresponding cells express HOXB9 and show a strong nuclear staining of the protein, while epiblast cells display a weaker homogeneously distributed staining.

### HOXB9 protein expression is restricted in mouse peri-gastrulating embryos

The localization of HOXB9 protein in the mouse was then evaluated from blastocyst implantation to post-gastrulating embryo stages, stages for which *in situ* hybridization data are available in the literature from E6.5 on [8, 12–14].

**Fig 5:**
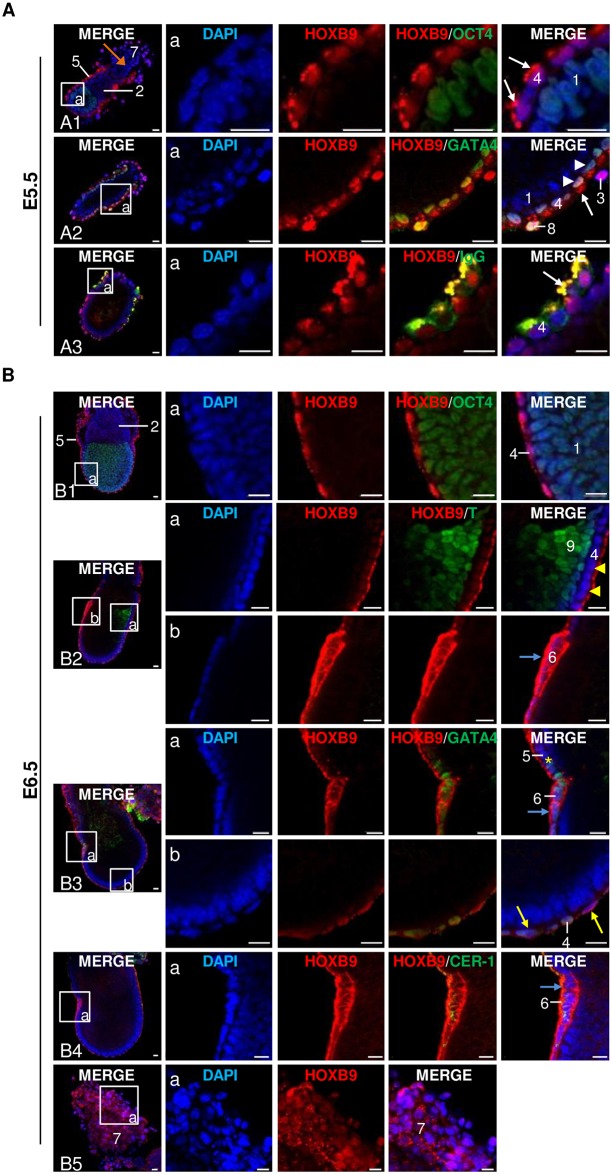
Mouse HOXB9 protein distribution in *in vivo* peri-gastrulating embryos: E5.5 and E6.5. **A-B:** Whole-mount immunofluorescence. **(A)** E5.5: HOXB9 proteins were co-stained with OCT4 **(A1)**, GATA4 **(A2)** or IgG **(A3)**. Arrows indicate HOXB9 that is localized in apical vacuoles of visceral endoderm cells. Orange arrows indicate the reduction of HOXB9 staining intensity observed in trophoblast cells in the center of the ectoplacental cone. Arrowheads indicate variation in HOXB9 nuclear staining between visceral endoderm cells. **(B)** E6.5: HOXB9 proteins were co-stained with OCT4 **(B1)**, BRACHYURY (**T—B2**), GATA4 **(B3)** and CER-1 **(B4)**. Zoom on the ectoplacental cone **(B5)**. Yellow arrows, arrowheads and the asterisk show the flattened, cuboidal and columnar cells of the visceral endoderm, respectively. Blue arrows indicate the strong HOXB9 staining observed in anterior visceral endoderm cells. 1. epiblast; 2. extra-embryonic ectoderm; 3. primary trophoblastic giant cells; 4. embryonic visceral endoderm; 5. extra-embryonic visceral endoderm; 6. anterior visceral endoderm; 7. ectoplacental cone; 8. parietal endoderm; 9. primitive streak. Nuclei: Blue; HOXB9: Red; OCT4/GATA4/IgG/T/CER-1: Green. Representative confocal Z-section. Scale bar = 20 μm.

**Fig 6:**
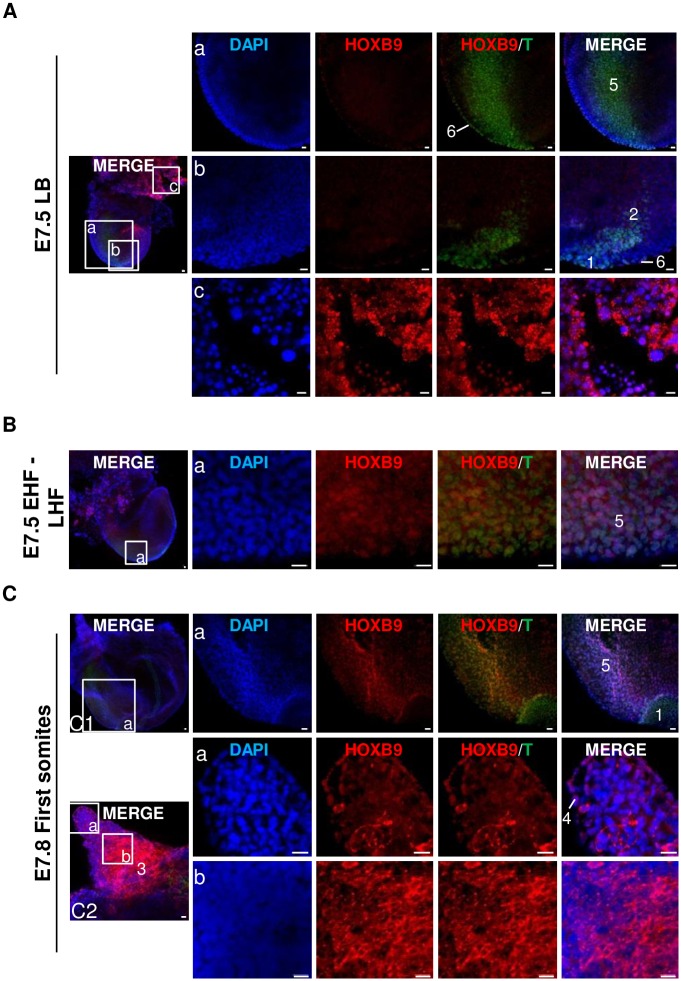
Mouse HOXB9 protein distribution in *in vivo* peri-gastrulating embryos: E7.5 and E7.8. **A-C:** Whole-mount immunofluorescence. **(A)** E7.5 late allantoic bud stage (LB). **(B)** E7.5 early headfold—late headfold stage (EHF—LHF). **(C)** E7.8 first somites stage. Zoom on allantois **(C2)**. 1. node; 2. notochord; 3. allantois; 4. mesothelium of the allantois; 5. primitive streak; 6. embryonic visceral endoderm. Nuclei: Blue; HOXB9: Red; T: Green. Representative confocal Z-section. Scale bar = 20 μm.

Mouse embryos produced *in vivo* were collected at E5.5, E6.5, E7.5 (LB and EHF—LHF stages, respectively) and E7.8 (first somites stage) and submitted to whole-mount immunofluorescence staining (Figs 5 and 6). At each stage, at least 3 embryos were handled in each experiment and the experiment was repeated at least twice.

At E5.5, HOXB9 displayed a strong nuclear localization in trophoblast cells located at the periphery of the ectoplacental cone (EPC), while the staining became progressively weaker and eventually disappeared in cells lying close to the center of the cone (Fig 5A1 –orange arrow). Extra-embryonic ectodermal (ExE) cells were also devoid of staining (Fig 5A1). Primary trophoblastic giant cells (TGCIs), derived from the mural TE cells undergoing endoreduplication [65], also presented a strong nuclear staining (Fig 5A2). Conversely, HOXB9 was barely detected in epiblast cells (Fig 5A1 –section a). Embryonic (EVE) and extra-embryonic visceral endoderm (ExVE), deriving from GATA4 positive PrE cells [66, 67], showed a strong punctuated HOXB9 staining at the apical pole of cells, likely corresponding to cytoplasmic vesicles (Fig 5A–arrows—see infra). Nuclear staining was weaker and displayed variable intensities in these cells (Fig 5A2—arrowheads). In parietal endoderm cells, also known to express GATA4 [66, 67], HOXB9 was abundant in both the nucleus and the cytoplasm, stained cytoplasmic vesicles showing no obvious polarity in their distribution (Fig 5A2).

Visceral endoderm is well-known to be a polarized epithelium [68, 69] that supplies maternal nutrients to early mammalian embryos [70]. During normal development in the mouse (but not in ruminants), IgGs are endocytosed by VE cells [71, 72] and accumulated in vesicles called apical vacuoles (a specialized type of lysosome—[21, 73, 74]). To determine whether HOXB9 staining co-localized with these apical vacuoles, a co-staining with mouse IgGs was performed on E5.5 embryos. Fig 5A3 (arrow) depicts a clear co-localization between HOXB9 and mouse IgGs at apical vacuoles.

At the onset of gastrulation (E6.5), the superficial trophoblastic cells of EPC displayed a heterogeneous pattern for HOXB9 staining (Fig 5B5). Indeed, cells with both cytoplasmic and nuclear HOXB9 staining as well as cells with nuclear staining only were observed. Cytoplasmic HOXB9 spots, which could correspond to cytoplasmic vesicles, were also observed. The ExE remained negative for HOXB9 staining (Fig 5B1), as did epiblast cells (except some peripheral cells) that were clearly marked with OCT4 (Fig 5B1 –section a). Cells from primitive streak, identified via BRACHYURY (T) expression [45, 75], were also negative for HOXB9 (Fig 5B2—section a). At this stage, EVE cells are squamous at the distal pole of the embryo, whereas they tend to be more cuboidal and columnar with a more vacuolated cytoplasm when localized close to ExVE [68, 76]. The flattened cells of VE presented both cytoplasmic and nuclear HOXB9 localizations (Fig 5B3—section b—yellow arrows), while the cuboidal (Fig 5B2—section a—yellow arrowheads) and the more columnar (Fig 5B3—section a—yellow asterisk) cells showed HOXB9 localization in apical vacuoles similar to the one observed in E5.5. Finally, some VE cells located at the embryonic side opposite to primitive streak (Fig 5B2—section a) showed strong cytoplasmic HOXB9 signal (Fig 5B2—section b and Fig 5B3—section a—blue arrow), as opposed to the remaining VE. We thus hypothesized that these cells corresponded to the anterior visceral endoderm (AVE). A co-staining was therefore performed with CER-1, a secreted protein expressed by AVE cells [45]. The HOXB9 cytoplasmic signal in these cells did indeed co-localized with CER-1, confirming HOXB9 expression in AVE (Fig 5B4).

At the LB stage, HOXB9 protein distribution was similar to that observed in E6.5 embryos (Fig 6A). Both the node (formed at the anterior end of primitive streak—[50]) and the forming notochord (mesodermal median axis under the epiblast—[32]) were negative with regard to HOXB9 staining (Fig 6A—section b).

At the EHF-LHF stages, changes in immunostaining only appeared at the level of primitive streak where HOXB9 started to be expressed mainly in nuclei (Fig 6B).

At E7.8, stage characterized by the presence of somites, HOXB9 staining was more apparent in cells of primitive streak compared to EHF—LHF stage (Fig 6C1). Moreover, signal associated to HOXB9 was also present in the allantois (Fig 6C2). HOXB9 subcellular localization was nuclear in inner cells of the distal part of allantois and in the mesothelium (Fig 6C2—section a). Conversely, the signal was more intense and mainly cytoplasmic in the inner cells of the proximal region (Fig 6C2—section b). HOXB9 expression in allantois was already seen in EHF—LHF embryos.

To summarize, from E5.5 to E7.8, HOXB9 present a spatially-restricted and dynamic protein pattern in mouse embryos. HOXB9 expression is limited to trophoblast, PrE and its derivatives, and allantois cells. Modifications in the subcellular localization are also observed. The most striking feature of HOXB9 expression being (1) the progressive disappearance of the staining in epiblast cells and its reappearance in primitive streak once gastrulation had well progressed at the EHF—LHF stage, (2) the localization of HOXB9 at apical vacuoles in VE cells, (3) the association of HOXB9 with vesicles in trophoblast cells and (4) the cytoplasmic distribution of HOXB9 in AVE.

### Distribution of bovine HOXB9 is ubiquitous in peri-gastrulating embryos and shares similarities with mouse HOXB9 distribution

In order to determine whether the mouse HOXB9 expression pattern was conserved in mammalian species around gastrulation, bovine embryos produced *in vivo* at stages D14 to D18 were examined. D14 (N = 2) and D17 (N = 2) embryos were co-stained with CDX2 to highlight trophoblastic cells [77], while D15 (N = 1) and D18 (N = 1) embryos were co-stained with VIMENTIN (VIM) and DLX3 to localize embryonic and extra-embryonic mesoderm cells [16, 52, 56] or trophoblastic cells [42], respectively.

**Fig 7:**
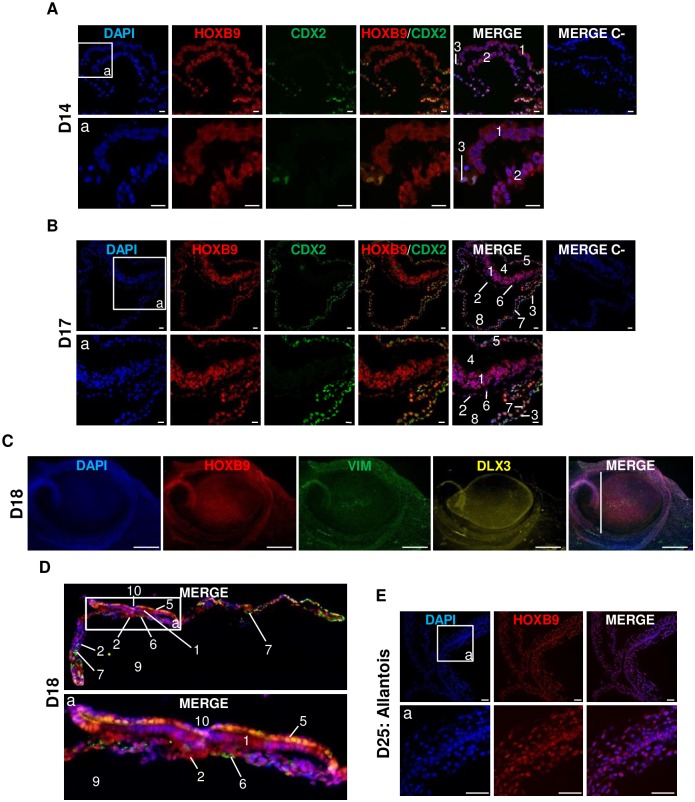
Distribution of bovine HOXB9 protein in *in vivo* peri-gastrulating embryos. **A, B:** Immunohistochemistry on D14 **(A)** and D17 **(B)** embryos. Merge C-: Negative control without primary antibody. Representative confocal Z-section. Scale bar = 20 μm. **C, D**: Whole-mount immunofluorescence on D18 embryos **(C)** followed by immunohistochemistry **(D)**. The localization of the sectioning is indicated by the white line in **C**. Epifluorescence images **(C)** and confocal Z-section **(D)**. Scale bar = 500 μm. **E:** Immunohistochemistry on allantois from D25 embryos. Representative confocal Z-section. Scale bar = 20 μm. Nuclei: Blue; HOXB9: Red; CDX2/VIMENTIN: Green; DLX3: Yellow. 1. epiblast; 2. primitive endoderm; 3. mural trophectoderm; 4. amniotic cavity; 5. amniotic wall; 6. embryonic mesoderm; 7. extra-embryonic mesoderm; 7 + 3 = chorion; 8. coelom; 9 yolk sac cavity; 7 + 2 = yolk sac wall; 10. primitive streak.

Contrary to the restricted expression observed in peri-gastrulation mouse embryos, distribution of HOXB9 was largely ubiquitous in the bovine (Fig 7). Embryos examined at D14-15 had initiated gastrulation. Their shape appeared tubular and they displayed a completely formed primitive endoderm, while polar TE cells had disappeared, as expected [31]. At this stage, despite the presence of HOXB9 in all tissues, it is interesting to note that epiblast cells displayed a weaker staining than TE cells and presented homogeneous distribution between cells, as observed in D11 embryos (Fig 7A). Moreover, HOXB9 was mainly localized in the nucleus of mural trophectoderm cells, although a fainter cytoplasmic staining was also present. Likewise, PrE cells shared a strong nuclear and weaker cytoplasmic HOXB9 immunoreactivity.

At stages D17-18, epiblast cells presented intense HOXB9 staining in the nucleus and fainter staining in the cytoplasm (Fig 7B–7D). Trophoblast cells remained well stained, as did PrE (extra-embryonic endoderm) cells. The amnion, which was positive for both CDX2 (Fig 7B) and DLX3 (Fig 7D), displayed the same HOXB9 pattern as trophoblast cells. The parietal extra-embryonic mesoderm was positive for HOXB9 and CDX2 as well (Fig 7B). Finally, all cells of the allantois of D25 embryos presented strong nuclear HOXB9 staining (Fig 7E), similar to that observed for the innermost cells of the distal part the mouse allantois.

To sum up, HOXB9 is ubiquitously expressed in the bovine embryos. The abundance of HOXB9 is reduced in epiblast cells of D14-15 embryos compared to trophoblast cells in a nearly homogeneous way between epiblast cells, as observed for D11 embryos. Thereafter, HOXB9 distribution evolves into the strong nuclear staining observed in D17-18 embryos. Similarly to the mouse embryos, HOXB9 expression is downregulated in bovine epiblast cells before gastrulation and again increase at later stages. In addition, the presence of HOXB9 proteins in TE, allantois and in PrE and its derivatives seems to be conserved between the two species.

## Discussion

**Fig 8:**
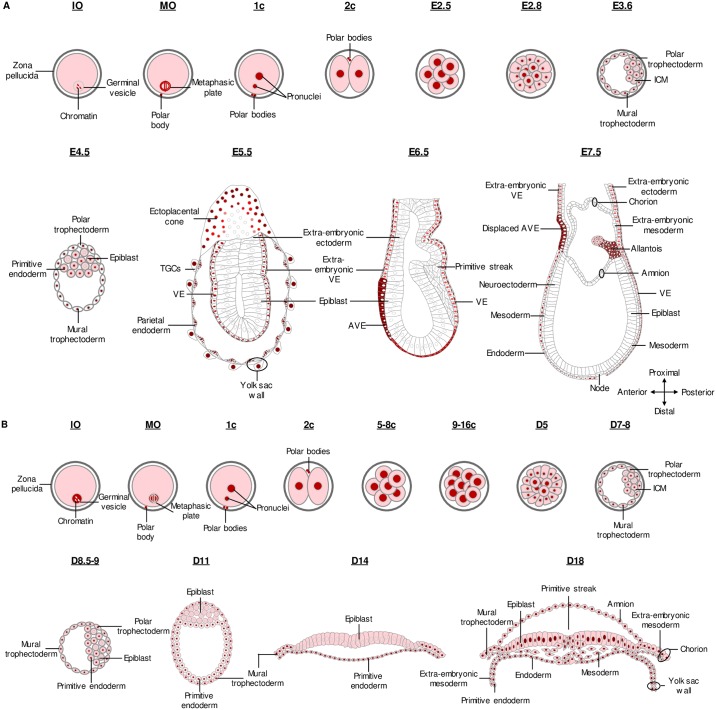
Schematic summary of the HOXB9 protein distribution in mouse (A) and bovine (B) early embryonic development. Immature oocyte (IO); Mature oocyte (MO); 1-cell embryo (1c); 2-cell embryo (2 c); 5- to 8-cell embryo (5–8 c); 9- to 16-cell embryo (9–16 c); Inner Cell Mass (ICM); Visceral endoderm (VE); Trophoblastic Giant Cells (TGCs); Anterior Visceral Endoderm (AVE). Tissues represented without nucleus in E7.5 embryos were not examined in details.

The general objective of this work was to study the localization of HOXB9 during oocyte maturation and early embryonic development (until peri-gastrulating stage) in mammals. Within this framework, we assessed for the first time the presence and the subcellular localization of the protein at specific time points during this period and performed a comparative analysis of HOXB9 distribution in the mouse and bovine. A schematic comparison of the subcellular distribution of HOXB9 in mouse and bovine oocytes and in the different cell types of early embryo until the peri-gastrulation period is shown in Fig 8. In summary, we demonstrated that (1) HOXB9 distribution is conserved in the two species in oocytes and from the zygote to the blastocyst stage, with a mainly nuclear localization; (2) at the blastocyst stage, in both species, nuclear HOXB9 is more abundant in TE than in ICM where its abundance decreases in a non-uniform way during blastocyst expansion; (3) in the bovine, a strong nuclear staining is observed in the forming PrE, while signal was concomitantly reduced in epiblast cells; (4) in mouse pre- and post-gastrulating embryos, HOXB9 is restricted to the trophoblast, PrE and derivatives, and allantois, presenting various surprising intra-cellular localization including the apical vacuoles of VE cells (from E5.5), the vesicles of trophoblast cells (from E6.5) and the cytoplasm of AVE cells; (5) during gastrulation, bovine epiblast cells re-acquire a strong HOXB9 signal in nuclei, while in the mouse, HOXB9, which was almost extinct in the epiblast, appears turned on in primitive streak.

Overall, throughout the developmental period studied, HOXB9 was found in the nucleus and cytoplasm. Of course, nuclear HOXB9 localization could be linked to the well described transcription factor role of HOXB9. However, HOXB9 could also be implicated in other processes taking place in the nucleus [7], such as DNA repair [78]. The cytoplasmic localization suggests possible non-transcriptional functions of the protein. In the cytoplasm, HOXB9 could act as translation regulator, as it has been demonstrated for HOXA9 [79]. Changes in HOXB9 distribution between the nucleus and cytoplasm could also reflect a way to regulate its transcriptional activity, as it has previously been reported for HOXA2 [80].

From the immature fully grown oocyte to the morula stage, HOXB9 protein was detected in all cells with a strong nuclear localization, both in the mouse and bovine. Our study is thus in accordance with previously published mRNA profiles showing the presence of maternal transcripts up to MET and *de novo* transcription from the embryonic genome afterwards [24]. However, our study could not differentiate between the maternal or embryonic origin of the protein. It is therefore not known if maternal HOXB9 is still present after MET, as already demonstrated for other proteins such as OCT4, CDX2 or SOX2 [81–83].

This period of development is marked by important events such as oocyte maturation, fertilization and EGA, which occurs between the 5- to 8-cell and the 9- to 16-cell stage in the bovine and before the 2-cell stage in the mouse [28, 84]. HOXB9 might thus play a role in the control of EGA but could also regulate the very few genes that are transcribed at the earliest stages. Several genes have been shown to be directly or indirectly regulated by HOXB9 [47, 85–87] and, for some of them, are known to be expressed in oocytes and/or early embryos and could potentially act downstream of HOXB9 in such context. For example, HOXB9 is known to directly regulate *TGF-β2* transcription and the TGF-β/SMAD pathway is crucial for the bidirectional communication between the oocyte and the surrounding granulosa/cumulus cells [88].

At the blastocyst stage, a progressive change was observed in HOXB9 subcellular localization in both species. While a strong nuclear staining was still observed in TE cells, the nuclear staining heterogeneously decreased between nuclei of ICM cells. This is in accordance with the study by Ozawa et al. [89], which reported a trend towards a greater *HOXB9* expression in TE cells compared to ICM cells in bovine blastocysts produced *in vitro*. Conversely, the study of Hosseini et al. [90] identified the *HOXB9* gene as exclusively expressed in ICM of bovine blastocysts produced *in vivo*. This discrepancy between *in vitro* and *in vivo* embryos remains difficult to reconcile all the more so as we observed the same pattern of HOXB9 protein distribution both in *in vivo* and in i*n vitro* blastocysts.

Since HOXB9 is mainly nuclear in TE, it is likely to act as a transcription factor during establishment and maintenance of TE. Interestingly, many direct or indirect HOXB9 target genes identified in distinct contexts [47, 85–87] are expressed in TE. AMPHIREGULIN is an EGF-like peptide involved in TE cell proliferation in the mouse and pig [91, 92]. In the pig, *AMPHIREGULIN* expression profile is similar to the one observed for the bovine *HOXB9* [92]. TGF-β2, another HOXB9 target, is detected in the mouse [93, 94] and pig [95] TE cells and is present in the fetal and maternal components of the bovine placentome [96, 97]. β-CATENIN and E-CADHERIN are present in the mouse and bovine from oocytes to blastocysts [98–100]. Together, they play a crucial role during morula compaction and TE formation [101–105]. *N-CAM* expression has been observed in bovine blastocysts (D. Paul and L. Bridoux, personal communication) and the protein, known to mediate cell adhesion, is present in murine TE cells [106]. Moreover, HOXB9 co-localized with CDX2, a validated *HOXB9* regulator involved in TE lineage determination [58, 107–109], in TE of both species and in a TE-derived bovine cell line (CT-1 cells–S1 File). Considering the co-localization of CDX2 and HOXB9 in TE cells, the impact of a *CDX2* knock-down on *HOXB9* mRNA in CT-1 cells was assayed (S1 File). *CDX2* knock-down in CT-1 cells did not appear to impact *HOXB9* mRNA relative expression.

Inner cell mass of E3.5 mouse and D7 bovine blastocysts is a mosaic of PrE and epiblast progenitors that will completely segregate at the late blastocyst stage. HOXB9 protein expression was heterogeneous in ICM of mouse (E3.6) and bovine blastocysts (from D7.5). However, this did not systematically correlate with the presence of the PrE markers (GATA6 or GATA4). In the bovine, once the PrE and epiblast segregation occurs, PrE cells strongly expressed HOXB9, while its expression was reduced but homogeneous in epiblast cells, suggesting an involvement of HOXB9 in bovine PrE formation. Although E4.5 mouse embryos analyzed in this study did not display a PrE, the detection of HOXB9 in PrE derivatives at later stages suggested a similar pattern in both species. Moreover, as was the case in the bovine, mouse HOXB9 protein was down-regulated in epiblast cells from E5.5 embryos.

The nuclear HOXB9 observed in TE cells persisted in trophoblast cells of both species at later stages. This observation is in accordance with *HOX* gene expression, including *HOX9*, reported in the mouse and human placenta [110–113]. In trophoblast cells, HOXB9 could contribute to the implantation process (reviewed in [114, 115]) by promoting the epithelial-to-mesenchymal transition (EMT), angiogenesis, cell migration and invasion (mouse) through the regulation of angiogenic factors and *TGF-β2*, as evidenced in cancers [47, 85–87]. Moreover, in a subset of superficial trophoblast cells from E6.5 mouse embryos, HOXB9 was observed in association with cytoplasmic vesicles. Trophoblast cells release exosomes that play important roles in the intercellular communication that contributes to placentation and to the development of maternal-fetal exchanges [116]. Furthermore, exosomes deriving from human cancer cells are shown to contain HOX proteins [117] and facilitate angiogenesis [118]. It is therefore possible that HOXB9 is released from trophoblast cells in exosomes and participates in the placentation process in this species. Due to the late placentation in the bovine (D21), no bovine trophoblast cells corresponding to the timing of implantation could be observed.

The localization of HOXB9 protein in mouse peri-gastrulating embryos and E12.5 embryos coincided with expression patterns determined by *in situ* hybridization, with the exception of the staining observed in PrE derivatives, which was not previously reported [8, 12–14]. Localization of HOXB9 in PrE derivatives was observed both in the mouse and bovine. Visceral endoderm influences the differentiation and development of blood islands and vessels in the underlying extra-embryonic mesoderm and coordinates blood cell differentiation [70]. HOXB9 could be involved in both processes, via the induction of angiogenic factors expression but also through an active role in embryonic erythropoiesis, as previously suggested [10].

Mouse HOXB9 co-localized with maternal IgGs within or associated to VE apical vacuoles at E5.5. Prior to the formation of a functional placenta in mouse embryo (at E10), nutrients as well as signaling molecules are endocytosed by VE cells and the resulting early endosomes deliver their content to apical vacuoles through microautophagy [119]. This process is essential for the embryo as shown by the developmental defects, particularly at the gastrulation step, observed in loss of function experiments of proteins involved in VE cell endocytosis [120–122]. On one hand, the presence of HOXB9 within VE apical vacuoles could reflect the presence of extracellular HOXB9 cooperating with signaling pathways to control the embryo patterning. Whether vacuole-associated HOXB9 is of maternal and/or embryonic origin has to be determined. On the other hand, if HOXB9 is associated with the cytoplasmic interface of apical vacuoles, HOXB9 could be originating from VE. In both situations, the association of HOXB9 with apical vacuoles might be linked to the modulation of signaling pathways and/or could represent a way of regulating HOXB9 activity.

A prominent HOXB9 staining was observed in the cytoplasm of mouse AVE, which is an early organizer that specifies the anterior-posterior axis of the embryo [70]. The cytoplasmic localization of HOXB9 in AVE could reflect a regulation of its transcriptional activity or its function as a translation regulator.

HOXB9 was expressed in the allantois in the mouse and bovine. Scotti and Kmita [123] previously demonstrated a *Hoxa10*-*Hoxa13* expression in the allantois of E7.5 and E8.5 mouse embryos, which is required for proper expansion of the fetal vasculature in the placenta. Ledgard et al. [15] and Hue et al. [16] also reported *HOX* expression in the bovine allantois. In the mouse, CDX2 was reported to be essential for allantoic growth and chorio-allantoic fusion [124]. HOXB9 could therefore act downstream of CDX2 in the control of allantois cell proliferation and in the development of a functional placenta.

As previously mentioned, *Hoxb9*^-/-^ mutant animals are viable and fertile indicating that maternal and zygotic *Hoxb9* loss of function is not embryo lethal. However, no exhaustive studies have so far been performed on *Hoxb9*^-/-^ mutant animals with regard to ovulation/maturation rates, littermate size or early development and implantation rates of their offspring. Moreover, the absence of a phenotype could be due to the presence of other HOX proteins, including HOX9 proteins, whose expression has previously been detected during early embryonic development [24], that could be functionally redundant. In addition, it is likely that HOXB9 acts in concert with other actors to control early embryonic development. Although not critical for oocyte/embryo development, as highlighted by loss of function studies, the involvement of maternal and/or zygotic HOXB9 in one or several of these processes cannot be ruled out. In order to address the function of both maternal and zygotic HOXB9, a knock-down approach by RNA interference in bovine oocytes, zygotes or trophectoderm cell line (CT-1 cells) was attempted (S1 File). Unfortunately, experiments did not significantly impact the protein abundance and conclusions on the role(s) maternal and zygotic HOXB9 could play in early embryonic development could not be drawn.

In conclusion, from the HOXB9 protein profile established in this exhaustive study, we could hypothesize a previously unsuspected involvement of HOXB9 in mammalian early embryonic development and, more specifically, in cell lineage differentiation, embryo patterning, allantois development and implantation.

## Supporting Information

S1 File HOXB9 functional analyses during bovine oocyte maturation and early embryo development. (DOCX)
