# Supplementary material for: Dynamic Pattern of HOXB9 Protein Localization during Oocyte Maturation and Early Embryonic Development in Mammals
Source: PLoS One. 2016 Oct 31;11(10):e0165898. doi: 10.1371/journal.pone.0165898 (PMC5087947; doi:10.1371/journal.pone.0165898)
Supplement: S1 File — (DOCX) [file pone.0165898.s001.docx]

**S1 File. HOXB9 functional analyses during bovine oocyte maturation and early embryonic development**

To investigate the function(s) HOXB9 could play during oocyte maturation and early embryonic development, a knock-down system by RNA interference was developed. Since *in vitro* maturation is poorly effective in the mouse species and biological material is more readily available in the bovine species, the functional study was conducted on this last one.

# Materials and methods

## Cell culture

The bovine trophectoderm cell line (CT-1) was kindly provided by N. Talbot (U.S. Department of Agriculture, Beltsville, USA - [1, 2]). CT-1 cells were cultured under 5 % CO_2_ at 37 °C in Dulbecco’s Modified Eagle Medium (DMEM) high glucose, pyruvate (Gibco, Gent, Belgium) supplemented with 10 % FBS (Invitrogen, Paisley, UK) and 0.2 % Primocin (Invivogen, San Diego, USA). For routine cell culture, cells were expanded in T75 flasks on collagen substrate (40 % PureCol® purified bovine collagen [#5005, Advanced Biomatrix, San Diego, USA], 5 % 0.1 M NaOH and 55 % CT-1 medium). Cells were physically recovered by scrapping followed by mechanical separation through an 18G needle. For transfection, RT-qPCR, immunocytochemical and western-blot analysis, cells were grown on CellBIND® multiple well plates (Corning, New-York, USA), as previously described [3].

## Immunostaining and imaging

For each immunostaining performed, a negative control without primary antibodies was involved.

### Whole-mount immunofluorescence

Bovine whole-mount immunofluorescence was performed on oocytes/embryos, as described in the article.

### Immunocytochemical analysis

CT-1 cells were seeded on 24-well plates (#3336, Corning) and were fixed for 20 min in 4 % PFA in PBS. Cells were permeabilized, blocked and incubated with antibodies, as described in the article. Primary and secondary used antibodies are listed in Table 1 and 2 of the article. Moreover, to detect CDX2 expression in CT-1 cells, a goat anti-mouse Alexa Fluor®555 secondary antibody (#4409, Cell Signalling, 1/500) was also used.

### Imaging

The labeled oocytes, embryos and cells were observed under a confocal microscope using the ZEN acquisition software (LSM710, Zeiss, Jena, Germany) or with an Axioskop 2 microscope.

## Western-blot

CT-1 cell proteins were extracted with RIPA buffer (50 mM Tris HCl pH 8, 150 mM NaCl, 1 mM EDTA, 1 % Nonidet P40, 0.5 % Na Deoxycholate, 0.1 % SDS) complemented with a protease inhibitor. Protein extracts were submitted to western-blot analyses, as described in the article.

Relative protein quantification was carried out using ImageJ software.

## Knock-down experiments by RNA interference in the bovine

Knock-down studies were carried out by RNA interference on bovine oocytes and embryos by dsRNA microinjection as previously described [4] and on CT-1 cells by dsRNA transfection. As a negative control, dsRNA with unrelated sequence (Scrambled - SCR) was used.

### dsRNA preparation

Three Dicer-substrate siRNA duplex oligonucleotides (DsiRNA) directed against *HOXB9* and two negative control duplexes (DS Scrambled negative control duplex and NC1 negative control duplex) were obtained from IDT (Tri*FECT*a kit, #BTC.RNAI.X001251896.12, Leuven, Belgium). A third negative control duplex was obtained from OriGENE (Trilencer 27 universal scrambled negative control siRNA duplex, #SR3004, Rockville, USA). A bovine *CDX2* 25-nucleotide Stealth siRNA duplex, previously designed by Schiffmacher and Keefer [5], was purchased from Life technologies (Gent, Belgium). Each of the three *HOXB9*-targetting duplexes and negative control duplexes were suspended in a RNAse-Free Duplex Buffer (IDT) to obtain a 100 µM solution, mixed in equal proportions and frozen until used. Considering *CDX2* knock-down experiments, 25-nucleotide Stealth siRNA duplex and NC1 Negative control duplex were diluted to obtain 20 µM and 10 µM solutions, respectively. dsRNA sequences are presented in Table A.

Table A. dsRNA sequences.

| dsRNA/Genbank accession number | Sequence | Length (nt) | Firm |
| --- | --- | --- | --- |
| HOXB9 #1  XM_001251896  NM_001191186.1 | 5’-CCCACAUACUUAUCCCAAAGUCACC-3’  3’-**AA**GGGUGUAUGAAUAGGGUUUCAGUGG-5’ | 27 | IDT |
| HOXB9 #2  XM_001251896  NM_001191186.1 | 5’-CCCGAGUACAGUUUGGAAACUUCGG-3’  3’-**GC**GGGCUCAUGUCAAACCUUUGAAGCC-5’ | 27 | IDT |
| HOXB9 #3  XM_001251896  NM_001191186.1 | 5’-AGAUGUAGUUGAUUCCUAAGAAGGT-3’  3’-**CA**UCUACAUCAACUAAGGAUUCUUCCA-5’ | 27 | IDT |
| CDX2 ^a^  NM_001206299.1 | 5’-AAAUUUUAACCUGCCUCUCUGAGAG-3’  3’-UUUAAAAUUGGACGGAGAGACUCUC-5’ | 25 | Life  technologies |
| Ds Scrambled negative control duplex | 5’-CUUCCUCUCUUUCUCUCCCUUGUGA-3’  3’-**AG**GAAGGAGAGAAAGAGAGGGAACACU-5’ | 27 | IDT |
| NC1 negative control duplex | 5’-CGUUAAUCGCGUAUAAUACGCGUAT-3’  3’-**CA**GCAAUUAGCGCAUAUUAUGCGCAUA-5’ | 27 | IDT |
| Trilencer 27 universal negative control siRNA duplex | 5’-CAUAUUGCGCGUAUAGUCGCGUUAG-3’  3’-**UG**GUAUAACGCGCAUAUCAGCGCAAUC-5’ | 27 | OriGENE |

Nt: nucleotide, ^a^ dsRNA from [5]. For a given gene, the first Genbank accession number refers to the sequence on which the dsRNA sequence was designed and the second one refers to a subsequent sequence update.

### *HOXB9* knock-down experiments in oocytes and zygotes

HOXB9 was knock-down in oocytes and zygotes following the protocol previously published [4]. Approximately 30 immature oocytes were manipulated at a time in TCM-199 containing 10 mg/ml of Polyvinylpyrrolidone (PVP). Part of the cumulus was removed by pipetting. A FemtoJet microinjection device (Eppendorf, Rotselaar, Belgium) was used to inject the dsRNA solution (7 pl, 70 hPa for 4 seconds). The microscope used for the microinjection was equipped with a heated plate. The outer diameter of the holding pipet was approximately 100 µm and the inner diameter was approximately 30 µm, as recommended by Favetta et al. [6]. Femtotips (Eppendorf) were used as injecting pipets. Approximately 80 zygotes free of cumulus cells were manipulated at a time in TCM-199 containing 0.4 mg/ml fraction V BSA. The same devices were used for injecting the cumulus-enclosed oocytes with adapted conditions (3.5 pl, 120 hPa for 2 seconds). Thereafter, immature oocytes were matured and zygotes were placed into culture droplets, as described in the article.

### *HOXB9* and *CDX2* knock-down experiments in CT-1 cells

CT-1 cells were seeded on 12 multiple well plates (#3336, Corning) on day 1 and were transfected after 2.5 days of culture. CT-1 cells were first rinsed with PBS and 400 µl of DMEM free of FBS and antibiotic were added. Per well, 3 µl of transfection agent (TransIT-siQUEST®, #MIR2110, Mirus, Madison, USA), warmed to RT and gently vortexed, were mixed to 100 µl of Opti-MEM® (#31985-032, Thermo Fisher Scientific, St Leon-Rot, Germany) containing siRNA duplex to reach a dsRNA concentration of 75 nM in the final volume of 500 µl (400 µl + 100 µl). After gentle vortexing, the mix was incubated for 25 min at RT and added to cells dropwise (100 µl). After 4 h of incubation at 37 °C, the medium was supplemented with FBS (10 %) and Primocin (0.2 %). Cells were then incubated O/N before complementing the medium with 500 µl of culture medium. Cells were harvested 20 h, 44 h and 68 h after addition of FBS and Primocin.

## RNA extraction, reverse transcription and quantitative PCR

### RNA extraction

Total RNA from denuded oocytes and embryos was extracted as previously described [7]. In brief, denuded oocytes and embryos were washed three times in nuclease-free PBS-PVP 0.2 g/l and frozen at -80 °C until use. Prior to extraction, 20 µg of glycogen (#AM9516, Glycoblue, Life technologies) were added as a carrier. Total RNA was extracted with 100 µl of TriPure isolation reagent (Roche, Mannheim, Germany) and 20 µl of chloroform. After precipitation with isopropanol, pellets were washed in 70 % ethanol, vacuum-dried and resuspended in 5 µl of nuclease-free water.

CT-1 cells were rinsed in PBS and harvested in 2 ml PBS. The cell suspension was submitted to 5 min centrifugation at 13,000g and the cell pellet was stored at -80 °C until RNA extraction. Total RNA was extracted using the High Pure RNA Tissue kit (#12033674001, Roche) following the manufacturer’s instructions.

### DNase treatment and reverse transcription

To eliminate genomic DNA contamination, total RNA was submitted to a DNase treatment according to the manufacturer’s instructions (#M6101, Promega, Madison, USA). RNA samples were then incubated at 65 °C for 10 min with 500 ng random hexamers (Roche) or 400 ng poly-dT (Roche) for oocytes/embryos or CT-1 cells, respectively, and reverse transcribed with a retrotranscription mix containing 50 U Expand Reverse Transcriptase (#11785834001, Roche), 1x buffer, 10 mM DTT, 1 mM dNTP (Roche) and 20 U RNaseOUT^TM^ (Life technologies) at 30 °C for 10 min followed by 45 min at 43 °C.

### Quantitative PCR

Primers were designed using the Primer Express® software (Applied Biosystems) based on NCBI database sequences (Table B). Quantitative PCR were run on an ABI Prism 7700 (Applied Biosystems) for oocytes/embryos knock-down experiments and on a StepOne plus (Applied Biosystems) for CT-1 cells knock-down experiments. Reaction mix consisted of 10 µl of Absolute qPCR SYBR Green mix (#AB-1163/B, Thermo Scientific), primers, 2 µl of diluted cDNA and nuclease-free water in a total volume of 20 µl. The qPCR protocol started with a 15 min step at 95 °C for enzyme activation followed by 40 cycles of denaturation at 95 °C for 15 seconds and a combined hybridization-elongation step at 60 °C for 1 min. A melting curve was generated to assess primers specificity. Each reaction was run in duplicate and a negative control containing no template was included for each gene. The mean of the quantification cycle (Cq) value was used to evaluate the relative abundance of the transcripts by the -ΔΔCq method [8]. Data were normalized with an internal normalization factor (ΔCq) based on three housekeeping genes selected with the GeNorm software (qbase+, Biogazelle, Gent, Belgium - [8, 9]): *GAPDH,* *H2A* and *YWHAZ* for oocytes/embryos knock-down experiments and *ACTG-1*, *YWHAZ* and *HPRT* for CT-1 cells knock-down experiments. For each replicate, a ratio was calculated with reference to negative control condition (ΔΔCq).

Table B. Primers for RT-qPCR.

| Gene | Sequence | Length (bp) | Trans-exon | [ ] (nM) |
| --- | --- | --- | --- | --- |
| *HOXB9^1^*  XM_001251896  NM_001191186.1 | F: 5’-GGACAATAAAATTTGCGAAGGA-3’  R: 5’-TGGTTTGATCCGGCCTCTC-3’ | 53 | No | 100 |
| *HOXC9^1^*  XM_002687231.2  XM_002687231.4 | F: 5’-ACAAAGAGGAGAAGGCCGACC-3’  R: 5’-AACGGGCGTGAATCCAGTTG-3’ | 51 | No | 300 |
| *CDX2*²  NM_001206299.1 | F: 5’-AGTCGCTATATCACCATCC-3’  F: 5’-CTTTCCTTTGCTCTGCG-3’ | 104 | Yes | 100 |
| *H2A^1^*  XM_005196640.1  XM_005196640.2 | F: 5’-AGAAGACGCGCATCATCCC-3’  R: 5’-ACTTTGCCCAGCAGCTTGTT-3’ | 79 | No | 250 |
| *GAPDH^1^*  XM_001252511.5 | F: 5’-TTCAACGGCACAGTCAAGG-3’  R: 5’-ACATACTCAGCACCAGCATCAC-3’ | 118 | Yes | 250 |
| *YWHAZ^1^*  XM_005215615.1  NM_174814.2 | F: 5’-GGTCATCTTGGAGGGTCGTCT-3’  R: 5’-CAGCACCTTCCGTCTTTTGC-3’ | 52 | No | 250 |
| *ACTG-1³*  NM_173979.3 | F: 5’-CCTCACGGAACGTGGTTACA-3’  R: 5’-TCCTTGATGTCACGCACAATTT-3’ | 87 | No | 200 |
| *HPRTI*  NM_001034035.2 | F: 5’-GGCTCGAGATGTGATGAAGGAG-3’  R: 5’-CAGAGGGCCACAATGTGATG-3’ | 51 | No | 200 |

F: forward, R: reverse, []: primer concentration. For a given gene, the first Genbank accession number refers to the sequence on which the primer sequence was designed and the second one refers to a subsequent sequence update.

^1^ Paul et al. [7]; ² Schiffmacher and Keefer [5]; ³ Goossens et al. [9].

## Statistical analysis

Western-blots quantification and RT-qPCR data from the knock-down experiments were analyzed using a Wilcoxon Signed Rank test (compared to 1). A Pearson chi square test was used to analyze the impact of *HOXB9* knock-down on the nuclear maturation. The effect of the knock-down in zygotes on the developmental rates was analyzed with an ANOVA2 test by comparing the HOXB9 dsRNA-injected embryos to their SCR-injected counterpart. Differences were considered significant at p-values lower than 0.05.

# Results

## Knock-down of bovine HOXB9 during oocyte maturation and early embryonic development

To gain an insight about the possible roles fulfilled by HOXB9 in the early embryo, we set up a siRNA-mediated gene inactivation in the bovine. Two strategies were developed, based on dsRNA injection in immature oocytes or in zygotes. The efficiency of the knock-down was first evaluated by RT-qPCR on *HOXB9* mRNAs 24 h post-injection for oocytes (6 replications). After injection of zygotes, *HOXB9* expression was evaluated 30 h post-injection (5- to 8-cell embryos – 7 replicates), 78 h post-injection (9- to 16-cell embryos – 3 replicates) and 150 h post-injection (blastocysts 7 dpi – 8 replicates). Expression of a closely related gene, *HOXC9*, was also quantified to check for the siRNA specificity (dsRNA injection in oocytes: 3 replicates, dsRNA injection in zygotes: 5 replicates). Injection of *HOXB9* dsRNA in immature oocytes induced a significant 61 % reduction of *HOXB9* mRNA levels (p = 0.016) in mature oocytes, as compared to scramble RNA (SCR)-injected negative control group (Fig 1A). Injection of dsRNA in zygotes led to a significant reduction of 70 % of *HOXB9* mRNA levels at the 5- to 8-cell stage (p = 0.004 - Fig 1B). This reduction was transient and was no longer observed in 9- to 16-cell embryos or in blastocysts. No significant effect was observed on *HOXC9* mRNA levels at any stage (Figs 1A and 1B), suggesting the specificity of the knock-down.

The impact of the *HOXB9* knock-down on protein abundance was next assessed by whole-mount immunofluorescence. We compared *HOXB9* dsRNA*-*injected, SCR-injected and non-injected oocytes at the mature oocyte and zygote stages, and dsRNA*-*injected, SCR-injected and non-injected zygotes at the 5- to 8-cell, 9- to 16-cell and blastocyst stages. Four to 15 oocytes or embryos of each stage (2 replicates) were evaluated in each condition. No clear difference was observed regarding HOXB9 protein abundance and distribution between the non-injected, the *HOXB9* dsRNA-injected and the SCR-injected oocytes/embryos at all analyzed stages (Fig 1C).


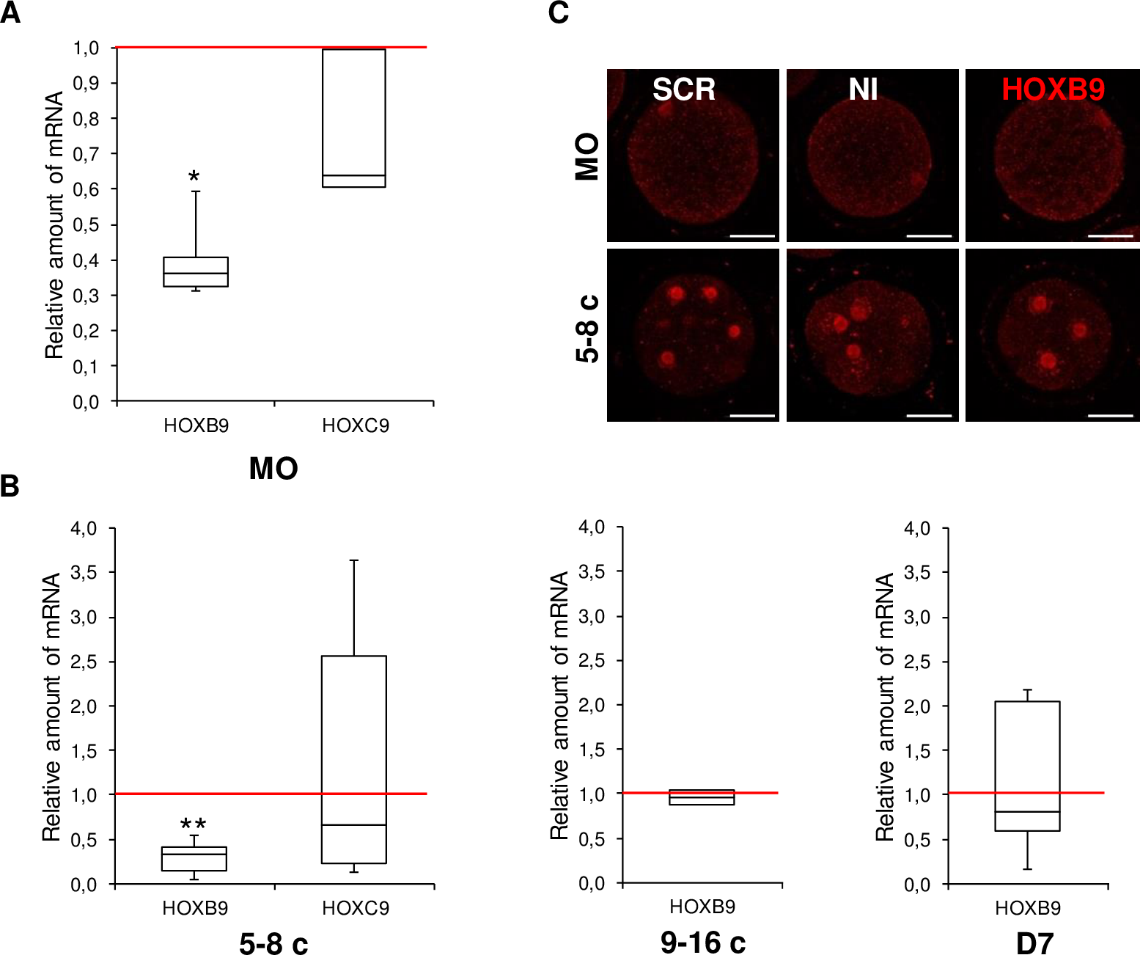


**Fig 1. Down-regulation of *HOXB9* expression during oocyte maturation and early embryo development.**

**A-B:** Relative quantification by RT-qPCR of *HOXB9* and *HOXC9* mRNA after injection of *HOXB9* dsRNA in oocytes **(A)** or in zygotes **(B)**. The ratio to the value in negative control dsRNA (SCR) transfected cells (SCR = 1 – red line) was calculated. The boxplots depict the distribution of the ratios. The ends of the whiskers represent the lowest or the highest datum. * Significant difference (Wilcoxon signed rank test, * = 0.01 < p < 0.05, ** = 0.001 < p < 0.01). Mature oocyte (MO); 5- to 8-cell embryo (5-8 c); 9- to 16-cell embryo (9-16 c) and blastocyst at day 7 post insemination (D7). **C:** Whole-mount immunofluorescence analysis of HOXB9 distribution in MO and 5-8 c embryos after no injection (NI), after injection of *HOXB9* dsRNA (HOXB9) or of SCR dsRNA (SCR) into immature oocytes or zygotes, respectively. Representative confocal Z-section. HOXB9: Red. Scale bar = 50 µm.

The effect of the *HOXB9* knock-down on nuclear maturation and developmental rates was addressed. Oocytes were stained with Hoechst 24 h post-injection (3 replicates – SCR: 78 oocytes, HOXB9: 66 oocytes). Injection of *HOXB9* dsRNA did not result in a significant effect on the number of oocytes reaching the metaphase II stage (SCR: 54 %; HOXB9: 49 %) and on proportion of embryos that cleaved (SCR: 64 %; HOXB9: 57 %), reached the 5- to 8-cell stage (SCR: 25 %; HOXB9: 25 %) or the D7 blastocyst stage (SCR: 21 %; HOXB9: 22 %). In a similar way, knocking-down *HOXB9* at the zygote stage (4 replicates – SCR: 385 zygotes, HOXB9: 315 zygotes) had no influence on the proportion of embryos that cleaved (SCR: 77 %; HOXB9: 85 %), reached the 5- to 8-cell stage (SCR: 50 %; HOXB9: 60 %) or the D7 blastocyst stage (SCR: 23 %; HOXB9: 30 %).

## siRNA-mediated *HOXB9* and *CDX2* knock-down in a bovine trophectoderm cell line (CT-1)

Due to difficulties encountered to impact bovine HOXB9 protein abundance in oocyte/embryo models, we attempted to inactivate *HOXB9* expression in an easier model. Since HOXB9 was highly expressed in TE cells all along the studied stages and to study HOXB9 function(s) in conditions as close as possible to physiological ones, we chose a bovine trophectoderm cell line (CT-1 cells). This cell line, derived from hatched D10-11 bovine blastocysts [1, 2], is regularly used to study trophoblast gene regulation [3, 5, 10-13] and allows evaluating HOXB9 function(s) in TE.

The trophectodermal nature of CT-1 cells was confirmed by evaluating by RT-PCR the expression of a combination of genes known to be characteristic of TE cells [10, 11]: *CDX2*, *IFNT* and *ERRB*. *HOXB9* expression was detected by RT-qPCR (3 replicates). The presence of HOXB9 and CDX2 proteins was checked by immunofluorescence (3 replicates - Fig 2A) and western-blot. HOXB9 and CDX2 presented a similar subcellular distribution. A nuclear staining was observed in all cells and the staining was diffuse in cells at the metaphase stage (Fig 2A - asterisk), as already observed in whole-mount embryos. The presence of CDX2 in CT-1 cells confirmed the results previously reported [5]. The impact of siRNA-mediated *HOXB9* knock-down on mRNA and protein abundance was then assessed by RT-qPCR and western-blot, 24 h, 48 h and 72 h post-transfection. Unfortunately, the decrease in *HOXB9* mRNA level was low, transient and poorly reproducible (from 0 % to 53 %) despite the use of several transfection agents. Furthermore, no impact was observed on HOXB9 protein abundance.

CDX2 has been identified as a regulator of *Hoxb9* expression in the mouse embryo [14]. Considering the co-localization of CDX2 and HOXB9 in trophectoderm cells, we therefore assayed the impact of a *CDX2* knock-down on *HOXB9* mRNA in CT-1 cells. Six samples from 3 repetitions were analyzed at each timing. A significant (p < 0.05) decrease in *CDX2* relative expression was observed after dsRNA transfection with 47 % relative decrease 24 h post-transfection, 37 % 48 h post-transfection and 35 % 72 h post-transfection (Fig 2B). This in turn led to a significant CDX2 protein abundance decrease, with the greatest protein diminution (76 %, p = 0.004 – 4 replicates, total of 8 samples analyzed) obtained 48 h post-transfection (Figs 2C and 2D). However, downregulation of *CDX2* did not significantly affect *HOXB9* mRNA relative expression, neither 24 h, 48 h nor 72 h post-transfection (Fig 2B).


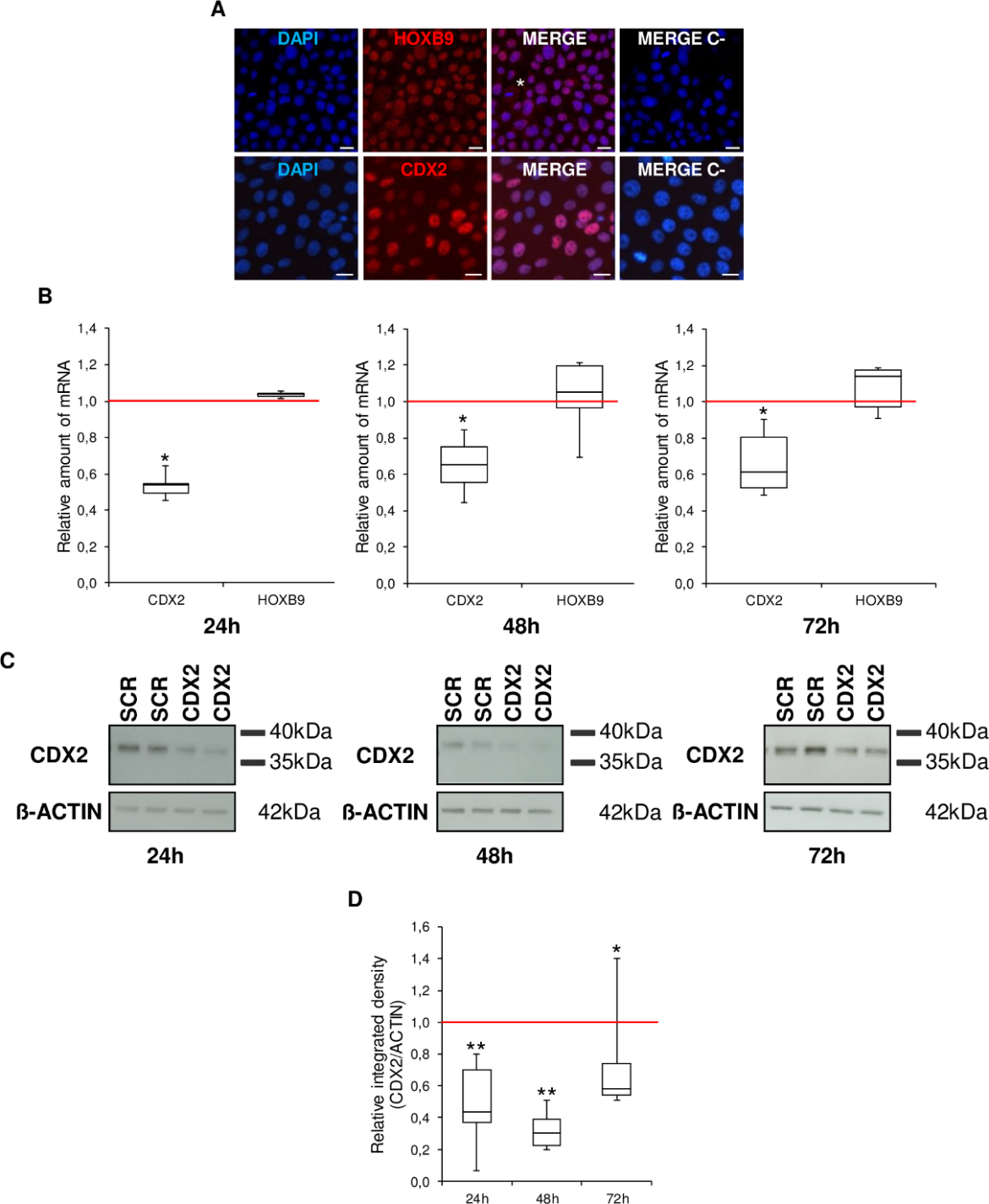


**Fig 2. Down-regulation of *CDX2* expression in CT-1 cells.**

**A:** HOXB9 and CDX2 detection by immunofluorescence in CT-1 cells. The asterisk indicates CT-1 cell in metaphase. Merge C-: negative control without primary antibody. Nuclei: Blue; HOXB9 or CDX2: Red. Representative confocal Z-section for HOXB9 and epifluorescence images for CDX2. Scale bar = 20 µm. **B:** Relative quantification by RT-qPCR of *CDX2* and *HOXB9* mRNA 24 h, 48 h or 72 h after *CDX2* dsRNA transfection in CT-1 cells. The ratio to the value in negative control dsRNA (SCR) transfected cells (SCR=1 – red line) was calculated. **C:** CDX2 proteins detection by western-blot in *CDX2* or SCR dsRNA transfected cells 24 h, 48 h and 72 h after transfection. **D:** Relative CDX2/β-ACTIN abundance in cells transfected with *CDX2* dsRNA compared to the same ratio in SCR dsRNA transfected set at 1 (red line). The boxplot depicts the distribution of the ratios. The ends of the whiskers represent the lowest or the highest datum. * Significant difference (Wilcoxon signed rank test, * = 0.01 < p < 0.05, ** = 0.001 < p < 0.01).

1. Talbot NC, Caperna TJ, Edwards JL, Garrett W, Wells KD, Ealy AD. Bovine blastocyst-derived trophectoderm and endoderm cell cultures: interferon tau and transferrin expression as respective in vitro markers. Biology of reproduction. 2000;62(2):235-47. PubMed PMID: 10642558.

2. Talbot NC, Powell AM, Camp M, Ealy AD. Establishment of a bovine blastocyst-derived cell line collection for the comparative analysis of embryos created in vivo and by in vitro fertilization, somatic cell nuclear transfer, or parthenogenetic activation. In vitro cellular & developmental biology Animal. 2007;43(2):59-71. doi: 10.1007/s11626-007-9013-9. PubMed PMID: 17570020.

3. Schiffmacher AT, Keefer CL. Optimization of a lipitoid-based plasmid DNA transfection protocol for bovine trophectoderm CT-1 cells. In vitro cellular & developmental biology Animal. 2012;48(7):403-6. doi: 10.1007/s11626-012-9525-9. PubMed PMID: 22810382.

4. Paul D, Sauvegarde C, Rezsohazy R, Donnay I. How to study HOX gene expression and function in mammalian oocytes and early embryos. Methods in molecular biology. 2014;1196:19-36. doi: 10.1007/978-1-4939-1242-1_2. PubMed PMID: 25151155.

5. Schiffmacher AT, Keefer CL. CDX2 regulates multiple trophoblast genes in bovine trophectoderm CT-1 cells. Molecular reproduction and development. 2013;80(10):826-39. doi: 10.1002/mrd.22212. PubMed PMID: 23836438.

6. Favetta LA, Madan P, Mastromonaco GF, St John EJ, King WA, Betts DH. The oxidative stress adaptor p66Shc is required for permanent embryo arrest in vitro. BMC developmental biology. 2007;7:132. doi: 10.1186/1471-213X-7-132. PubMed PMID: 18047664; PubMed Central PMCID: PMC2220003.

7. Paul D, Bridoux L, Rezsohazy R, Donnay I. HOX genes are expressed in bovine and mouse oocytes and early embryos. Molecular reproduction and development. 2011;78(6):436-49. doi: 10.1002/mrd.21321. PubMed PMID: 21567651.

8. Vandesompele J, De Preter K, Pattyn F, Poppe B, Van Roy N, De Paepe A, et al. Accurate normalization of real-time quantitative RT-PCR data by geometric averaging of multiple internal control genes. Genome biology. 2002;3(7):RESEARCH0034. PubMed PMID: 12184808; PubMed Central PMCID: PMC126239.

9. Goossens K, Van Poucke M, Van Soom A, Vandesompele J, Van Zeveren A, Peelman LJ. Selection of reference genes for quantitative real-time PCR in bovine preimplantation embryos. BMC developmental biology. 2005;5:27. doi: 10.1186/1471-213X-5-27. PubMed PMID: 16324220; PubMed Central PMCID: PMC1315359.

10. Sakurai T, Bai H, Konno T, Ideta A, Aoyagi Y, Godkin JD, et al. Function of a transcription factor CDX2 beyond its trophectoderm lineage specification. Endocrinology. 2010;151(12):5873-81. doi: 10.1210/en.2010-0458. PubMed PMID: 20962045.

11. Sakurai T, Bai H, Bai R, Sato D, Arai M, Okuda K, et al. Down-regulation of interferon tau gene transcription with a transcription factor, EOMES. Molecular reproduction and development. 2013;80(5):371-83. doi: 10.1002/mrd.22171. PubMed PMID: 23606646.

12. Bai H, Sakurai T, Someya Y, Konno T, Ideta A, Aoyagi Y, et al. Regulation of trophoblast-specific factors by GATA2 and GATA3 in bovine trophoblast CT-1 cells. The Journal of reproduction and development. 2011;57(4):518-25. PubMed PMID: 21606631.

13. Xie M, McCoski SR, Johnson SE, Rhoads ML, Ealy AD. Combinatorial effects of epidermal growth factor, fibroblast growth factor 2 and insulin-like growth factor 1 on trophoblast cell proliferation and embryogenesis in cattle. Reproduction, fertility, and development. 2015. doi: 10.1071/RD15226. PubMed PMID: 26304178.

14. van den Akker E, Forlani S, Chawengsaksophak K, de Graaff W, Beck F, Meyer BI, et al. Cdx1 and Cdx2 have overlapping functions in anteroposterior patterning and posterior axis elongation. Development. 2002;129(9):2181-93. PubMed PMID: 11959827.
